# An integrative review of *APOL1* kidney disease with a focus on the Brazilian population

**DOI:** 10.1016/j.clinsp.2026.101109

**Published:** 2026-09-07

**Authors:** Celia M. Barbosa de Souza, Bibiana S. de Oliveira Fam, Giovanna C. Giudicelli, Precil Diego Miranda de Menezes Neves, Francisco V. Veronese, Fernanda S. Luiz Vianna

**Affiliations:** a Nephrology Service, Hospital de Clínicas de Porto Alegre (HCPA), Universidade Federal do Rio Grande do Sul (UFRGS), Porto Alegre, RS, Brazil; b Graduate Program in Medicine: Medical Sciences, Universidade Federal do Rio Grande do Sul (UFRGS), Porto Alegre, RS, Brazil; c Laboratory of Genomic Medicine, Experimental Research Center, Hospital de Clínicas de Porto Alegre (HCPA), Porto Alegre, RS, Brazil; d Laboratory of Immunobiology and Immunogenetics, Programa de Pós-Graduação em Genética e Biologia Molecular (PPGBM), Department of Genetics, Institute of Biosciences, Universidade Federal do Rio Grande do Sul (UFRGS), Porto Alegre, RS, Brazil; e Department of Genetics and Evolutionary Biology, Institute of Biosciences, Universidade de São Paulo (USP), São Paulo, SP, Brazil; f Division of Nephrology, Hospital das Clínicas, Faculdade de Medicina, Universidade de São Paulo (USP), São Paulo, SP, Brazil; g Center for Nephrology and Dialysis, Hospital Alemão Oswaldo Cruz, São Paulo, SP, Brazil; h INAGEMP, National Institute of Populational Medical Genetics, Porto Alegre, RS, Brazil

**Keywords:** Renal disease, *APOL1*, G1/G2 risk alleles, Brazilian Afro-descendants, Public health

## Abstract

Chronic Kidney Disease (CKD) has a global impact on morbidity and mortality, affecting disproportionately Afro-descendants with a risk of 4 to5 times higher compared to non-Afro-descendants. The increased risk is driven by both socioeconomic and biological factors, highlighting the role of *APOL1* as a pragmatic example of this scenario. Carriers of the high-risk genotype (two risk alleles) are approximately three times more likely to develop CKD and experience more severe disease progression. Despite the high impact on Afro-descendant health, few studies of APOL1 kidney disease investigated the impact in admixed populations. In this integrative review, we evaluate *APOL1* kidney disease globally, with a focus on the Brazilian population. We conducted a comprehensive search on PubMed, Lilacs, and Scielo databases. Publications in English, Spanish, and Portuguese were included, covering the period from the identification of *APOL1* G1 and G2 variants in 2010 through October 2025. The initial search found 831 articles, after screening and full-text assessment based on predefined inclusion criteria which only includes original research on African or Afro-descendant populations, 306 articles were included and analyzed. In total, ten Brazilian articles were identified. Brazilian studies highlighted the prevalence of *APOL1* risk variants, revealing the presence of G1 and G2 alleles in Afro-Brazilians and their significant role in the onset of CKD, lupus nephritis, and early kidney replacement therapy. The limited number of studies conducted within the Brazilian population underscores an urgent need for further research to tailor strategies in public health policies of *APOL1* kidney disease on Afro-Brazilian and admixture populations.

## Highlights

• CKD remains a major health burden in Latin America and middle-income regions.
• *APOL1* kidney disease poses diagnostic and therapeutic challenges in admixed populations.
• Studies on *APOL1* kidney disease are scarce in admixed and Afro-Brazilians.
• Findings support new public health strategies for Afro-Brazilian and admixed populations.

## Graphical abstract

**Figure:**
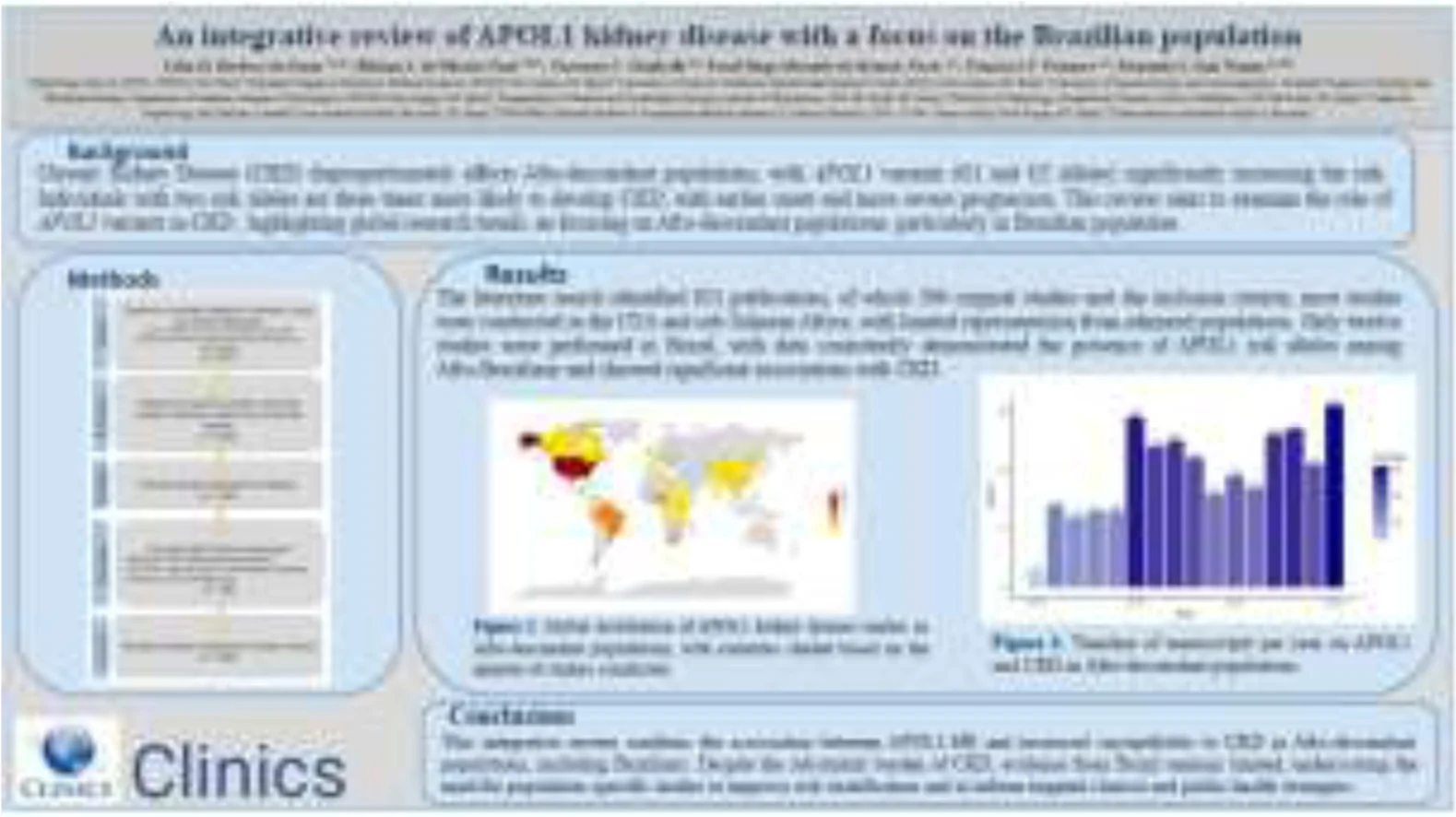

## Introduction

Chronic Kidney Disease (CKD) is a non-communicable disease of global importance, ranging from asymptomatic forms to Chronic Kidney Diseases requiring Kidney Replacement Therapy (CKD-KRT).^1^^,^^2^ In recent decades, the discovery of high-risk variants in the Apolipoprotein 1 gene (*APOL1*) significantly impacts the understanding of CKD progression among individuals with recent African ancestry. High-risk *APOL1* genotypes (G1 and G2) have been consistently associated with non-diabetic CKD, and increased susceptibility to adverse outcomes in various conditions, including hypertension, Focal Segmental Glomerulosclerosis (FSGS), HIV-Associated Nephropathy (HIVAN), and lupus nephritis.^3^ Furthermore, among Afro-descendants kidney transplant recipients, the presence of two risk alleles induces cytotoxicity and progressive kidney damage.^4^ This acknowledgment transforms primary healthcare approaches, and justifies the need to address diagnostic and treatment strategies for the earlier identification and management of this patient group.

Despite a general incidence of approximately 15% in the United States of America (USA), CKD presents a clear disparity.^5^ In 2023, the prevalence of CKD-KRT was over four times higher in Afro-descendants than in individuals with non-recent African ancestry.^6^ It is estimated that CKD has an incidence of >10% of the world's population, particularly affecting individuals living in low and middle-income countries, where limited access to healthcare hinders diagnosis, prevention, and treatment.^7^ In Brazil, a country with a highly admixed population^8^ and a significant proportion of self-declared Black and Brown individuals, these disparities require attention for addressing the risk and progression of APOL1 kidney disease. According to the DATASUS, the average national hospital mortality rates for kidney failure over the past decade were 13.29 (ranging 12.08‒14.59) per 100,000 inhabitants.^9^ The Brazilian National Dialysis Registry (2000‒2012) reported that 40.5% of patients on a chronic dialysis self-identified as Afro-descendants, while the National Survey by Continuous Household Sample (PNAD, 2012‒2019) reported that 56.2% of the total Brazilian population self-declared as Black or Brown.^10^ The 2022 Brazilian Dialysis Census highlights the growing prevalence and incidence of CKD-KRT, with rates of 758 per million population (pmp) and 214 pmp respectively.^11^ For this period an increase in the mortality rate was estimated at 20.3% among CKD patients, influenced by age, comorbidities, and the impact of COVID-19 pandemic.^12^

In Brazil, the estimated prevalence of CKD in adults is 6.7%, with a value that can reach up to six times higher in individuals aged 60 or older.^13^ Brazilian studies have reported different rates among regions in the country, with higher rates of CKD (∼3%) in the Northeast state of Bahia, particularly among elderly and male individuals. In contrast, other studies have reported that prevalence rates decreased over time, from 2.87% in 2003 to 1.43% in 2013.^14^ These discrepancies reflect influences of biological, sociodemographic, and regional factors. Despite this, research addressing the role of *APOL1* risk variants in the Brazilian population remains scarce. To address this gap, we conducted a comprehensive review of the scientific literature on this issue from the past 15 years, focusing on the prevalence and the impact of *APOL1* risk alleles on Afro-Brazilians, and their role in the risk of developing CKD.

## Methodology

The study was conducted through an integrative literature review based on an active search for scientific articles in the Pubmed, Lilacs, and Scielo online databases, accessed from August 2022 to October 2025. The search included articles on the variability of *APOL1* gene and its role in susceptibility to the development of CKD in Afro-descendant populations. The search strategy used the following syntax across all databases: ((chronic *AND* (kidney *OR* renal) *AND* disease) *OR* (glomerulosclerosis *OR* (glomerular *AND* sclerosis)) *OR* nephropathy) *AND* (*APOL1*). A time frame of 15-years was established, beginning in 2010, the year in which the *APOL1* high-risk genotype (APOL1-HR) was first described, and extending through October 2025. Individuals carrying two *APOL1* risk alleles (G1/G1, G2/G2, or G1/G2) were classified as APOL1-HR, whereas those carrying one risk allele (G0/G1, or G0/G2) are referred to as having a low-risk genotype (APOL1-LR).

**Fig. 1:**
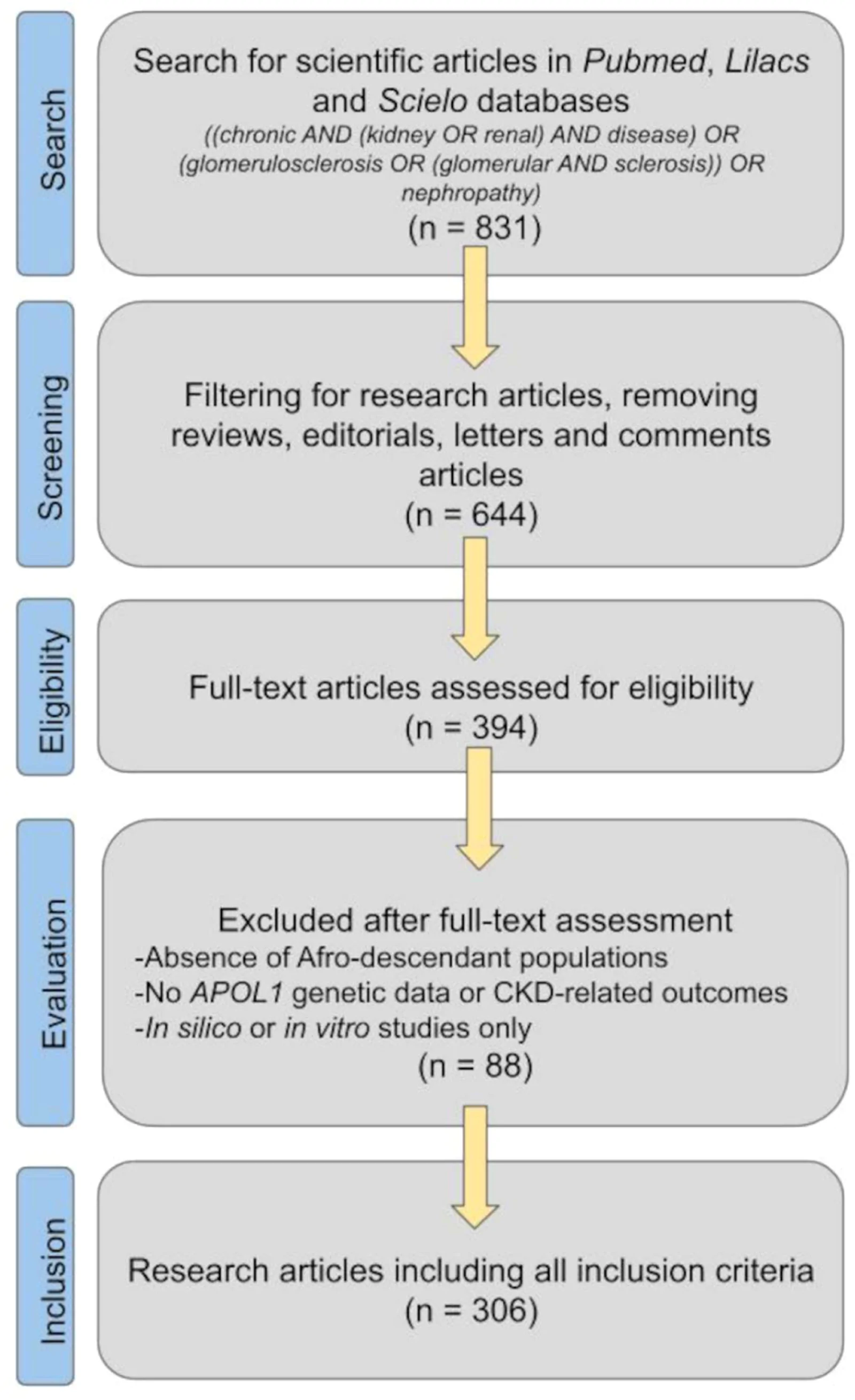
Flowchart describing the methodology used in the integrative search for bibliographic references in public databases from the key terms and the filtering criteria used to categorize the studies found.

All retrieved articles were screened according to predefined inclusion and exclusion criteria (Fig. 1). Inclusion criteria comprises original studies focusing on the association between *APOL1*-HR, CKD, and Afro-descendant populations. Exclusion criteria comprises articles unrelated to *APOL1*, kidney disease, or Afro-descendant cohorts. Additionally, publications reporting data on the Brazilian population were to provide contextual information on *APOL1* variants in Brazil, however, only original studies reporting primary data on *APOL1* risk variants and kidney disease were included. The quality of references from the public database was verified by organizing them into a comprehensive database. Key information was meticulously cataloged, including the title, first author, co-authors, PMID, DOI, publication date, study location, and main findings. To ensure accuracy, the list of references was manually validated by cross-checking the title and abstract of each selected article, excluding those unrelated to *APOL1*, kidney disease, or Afro-descendants. This integrative review was conducted in accordance with the PRISMA (Preferred Reporting Items for Systematic Reviews and Meta-Analyses) guidelines. Ethical considerations: Since this review was based on published manuscripts, there is no need for analysis by the Research Ethics Committee, according to Brazilian regulations.

## Results

**Fig. 2:**
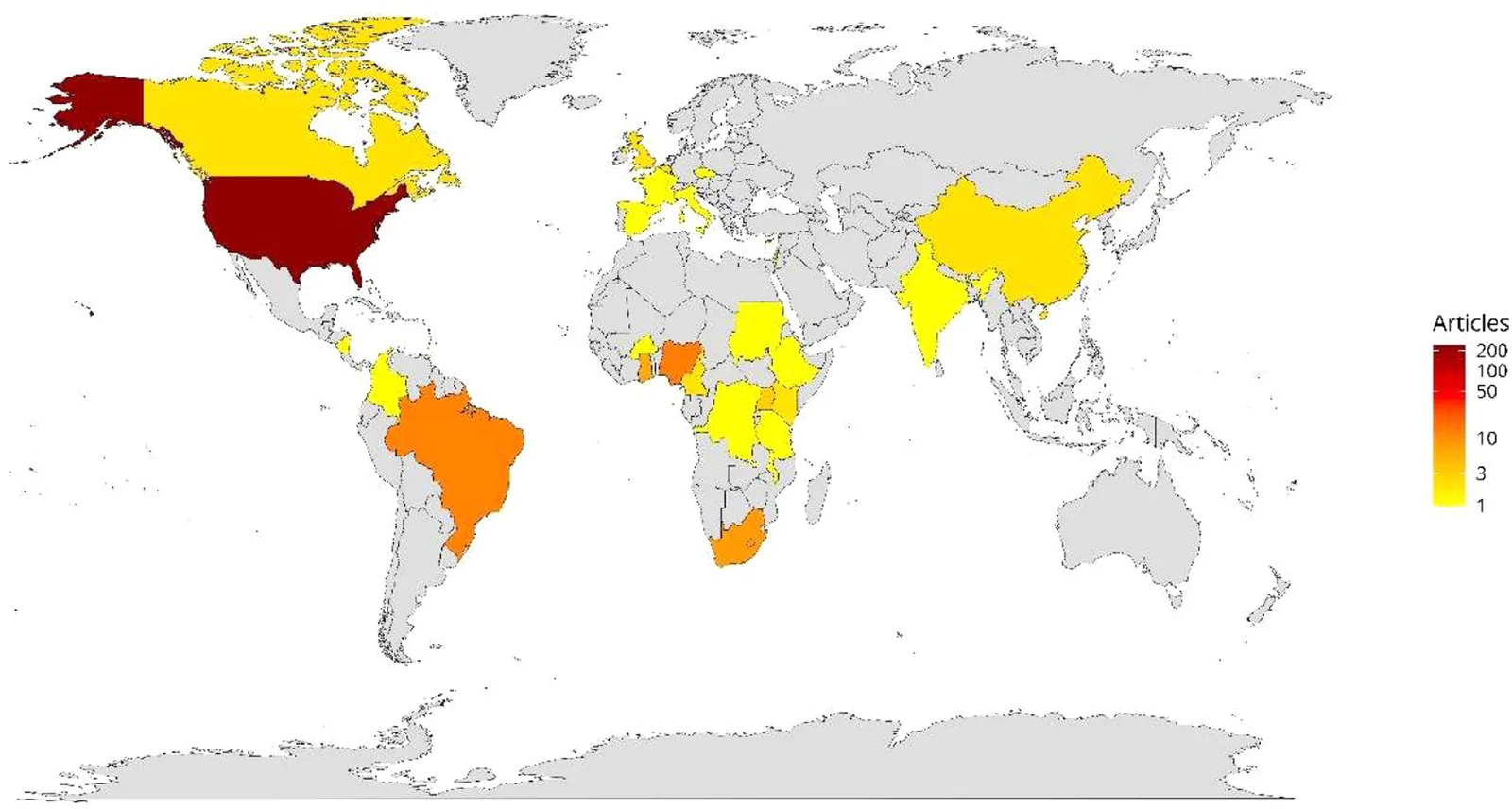
Global distribution of APOL1 kidney disease studies in Afro-descendant populations with countries shaded according to the number of studies conducted. The color gradient ranges from yellow, representing countries with the fewest studies, to dark red, representing countries with the highest number. Gray areas indicate regions with no data available. The visualization highlights geographic disparities in research contributions, emphasizing areas of higher and lower research focus.

**Fig. 3:**
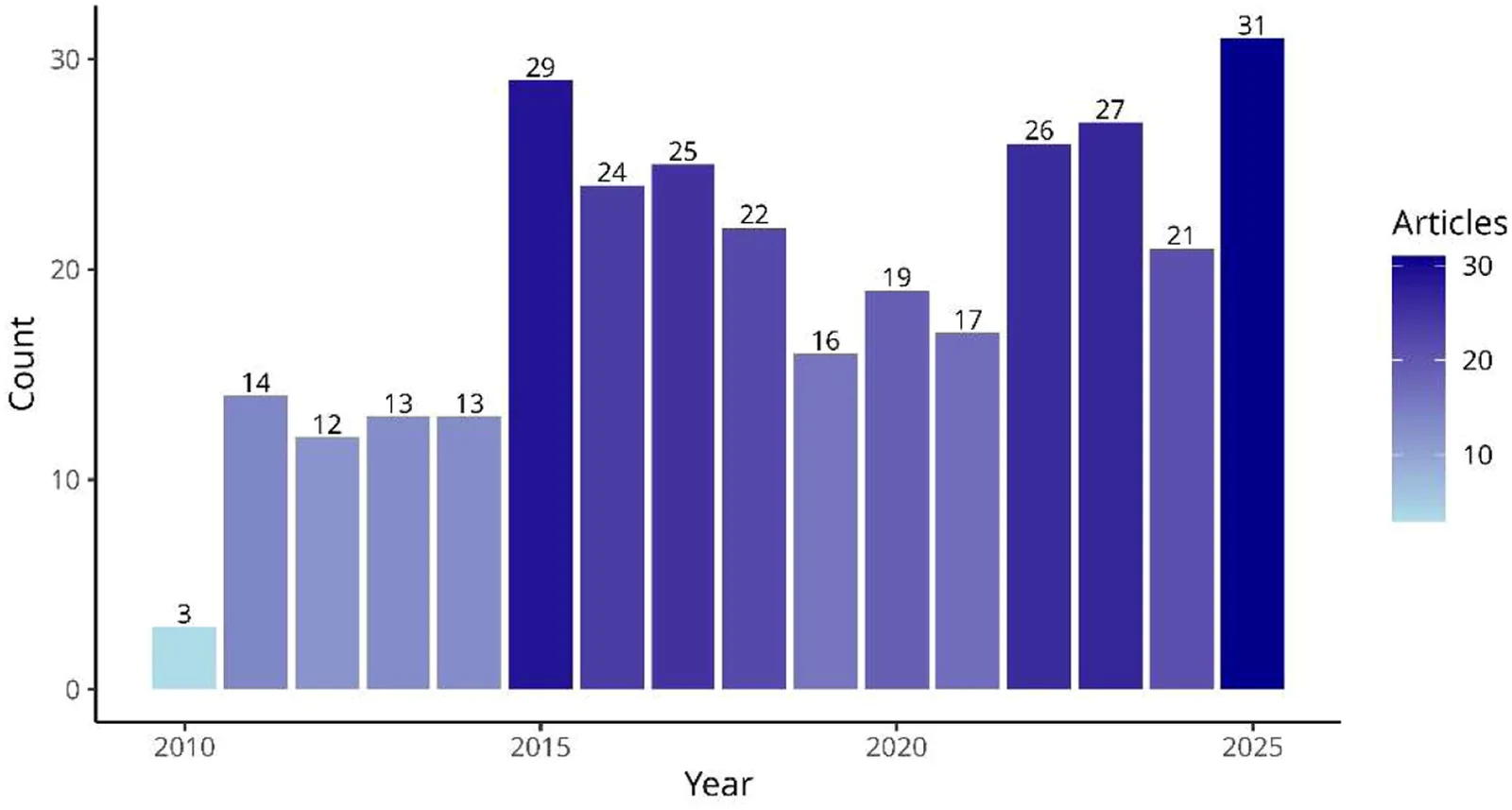
Graphic representation of the timeline showing the number of manuscripts published per year on APOL1 and Chronic Kidney Disease (CKD) in Afro-descendant populations from 2010 to 2025.

A total of 831 articles were initially retrieved across all databases analyzed. No unique records were identified in the Lilacs database, while two articles were found in the Scielo database, duplicated Pubmed results. After initial screening (Fig. 1), 187 articles were identified as reviews, leaving a total of 644 articles for further analysis. Of these, 250 articles were excluded for not meeting the inclusion criteria, as they were classified as commentaries, letters, or lacked original data. This process resulted in 394 articles eligible for full-text assessment. From this subset, 88 additional articles were excluded due to using only *in silico* or in vitro data with the absence of data concerning Afro-descendant populations. Finally, a total of 306 articles met the criteria for inclusion in this review (Table S1). These studies provided crucial insights into the relationship between *APOL1* genetic variants and the risk of developing CKD in Afro-descendant populations globally (Fig. 1). Analysis of the geographic distribution (Fig. 2) revealed that 33 articles focused on Africa, 5 on Asia, 2 on Central America, 9 on Europe, 244 on North America, and 13 on South America (Table 1). Among the South American studies, ten original research articles reported primary data on *APOL1* genetic variability in Brazilian populations, which are summarized in Table 2. In 2010, the year of the first description of the *APOL1* variants, fewer than five articles were published. Following the identification of the G1 and G2 variants, the number of publications increased, exceeding 10 publications per year, with an average of >16 articles annually (Fig. 3).

**Table 1:** Number of publications by year and geographical region on *APOL1* and CKD in African and African descendant populations from 2010 to 2025 providing an overview of the global distribution of research on this topic.

Publication Year	Africa	North America	Asia	Central America	South America	Europe	Total
2010	1	2	0	0	0	0	**3**
2011	0	14	0	0	0	0	14
2012	1	10	1	0	0	0	12
2013	3	9	1	0	0	0	13
2014	0	11	0	1	1	0	13
2015	2	27	0	0	0	0	29
2016	0	23	0	0	0	1	24
2017	2	21	2	0	0	0	25
2018	1	19	0	0	3	0	22
2019	4	12	0	0	1	0	16
2020	3	14	0	0	1	1	19
2021	2	13	0	0	1	1	17
2022	6	17	0	0	1	2	26
2023	1	23	0	1	1	1	27
2024	5	13	0	0	2	1	21
2025	2	16	1	0	4	2	25
**Total**	33	244	5	2	15	9	**306**

**Table 2:** Summary of studies investigating the relationship between *APOL1* high-risk genotypes and kidney disease in the Brazilian population. The table summarizes information as study type, participants numbers, reported effect size (HR) for *APOL1* high-risk genotypes, and the main findings.

Year	Region/State	Type of Study	Number of Patients	Cases/Controls	Sex	Ethnicity	OR (HR Genotype)	Frequency of HR Genotype	Summary of Results	Reference
2014	Brazilian data	Cohort	196	‒	F	35% Black or Brown, 65% White	‒	‒	MYH9, not APOL1, is associated with CKD in Lupus Nephritis patients regardless of race	Colares et al., ^32^
2019	Brazil (Paraná, Alagoas)	Case-control	274	136/138	M, F	Afro-Brazilians	OR = 10.95 [SE = 1.49]	APOL1-HR 9.4% in CKD-KRT vs. 0.9% in controls	Carriers of APOL1-HR started dialysis 12 years earlier; APOL1-HR show 10 times more common in CKD-KRT cases	Riella et al., ^27^
2020	Brazil (Pernambuco, São Paulo)	Case-control	423	201/222	M, F	Admixed population	‒	‒	APOL1-HR not significant in non-white LN cases; associated with CKD progression	Vajgel et al., ^31^
2021	Brazil (São Paulo)	Cohort	318	‒	M, F	210 White, 78 Pardo, 26 Black	‒	APOL1-HR 6%	APOL1-HR in the NS cohort increased CKD likelihood: 89% vs. 52%, p = 0.001	Watanabe et al. ^33^
2024	Brazil (São Paulo)	Longitudinal	70	‒	M, F	Admixture population	‒	80.5% HRG	APOL1-HR in Idiopathic collapsing glomerulopathy, 80.5% of patients, 50% progressed to CKD-KRT within 36 months	Neves et al., ^37^
2024	Brazil (São Paulo)	Cross-sectional	101	‒	M, F	Admixture population	‒	‒	APOL1-HR were identified in 8% of families and causative Mendelian variants in 12%	Watanabe et al. ^39^
2025	Brazil	Retrospective cohort	94	‒	M, F	Admixed population	‒	‒	CG patients 18/94 (19%) genotyped, FSGS & MCD linked to better renal outcome than TMA	Neves et al. ^44^
2025	Brazil (São Paulo)	Case report	1	‒	F	Admixed population	‒	‒	Patients with SLE and Parvovirus B19 developed collapsing glomerulopathy, improved after steroids. illustrates infection/immune “second hit"	Bernardes et al., 2025 ^36^
2025	Brazil (Rio Grande do Sul)	Case-control	485	‒	M, F	Admixed with African ancestry	OR = 4.59; 95% CI 1.41–14.96 for G1 allele	G1: 2.7%; G2: 1.2%	Carrying one APOL1 G1 allele was associated with cardiovascular complications during COVID-19 independent of CKD; risk alleles remained prevalent despite high European ancestry.	Cadore et al. ^43^
2025	Brazil (Rio Grande do Sul)	Cross-sectional	175	‒	M, F	Afro-Brazilians	‒	G1: 15.3%; G2: 5.9%	High frequencies of APOL1 risk alleles associated with CKD severity in Afro-Brazilians; ancestry analysis confirmed significant African component.	Souza et al. ^39^

Brazilian studies consistently highlighted an increased risk for FSGS, hypertensive nephropathy, and HIVAN in Afro-descendants carrying the G1/G2 high-risk variants, as well as a notably high frequency of *APOL1* risk alleles in the Brazilian population. To provide a comprehensive overview of the current knowledge, this review explores the role of *APOL1* in kidney disease, and the following sections address its association with CKD related to each condition. Furthermore, we evaluated studies involving Brazilian data discussing their implications for understanding *APOL1* kidney disease in Afro-descendant and admixed populations.

### Mechanisms of APOL1 mediated kidney toxicity and disease pathogenesis

The *APOL1* is one of the six apolipoprotein genes located on chromosome 22. It is estimated that the G1 and G2 alleles of *APOL1* originated approximately 4000-years ago in Africa, and their rapidly increasing frequency reflects positive selection driven by protection against *Trypanosoma* sp. infection (sleeping sickness).^15^ The G1 allele (rs73885319/A>G and rs6091014/T>G) is characterized by two non-synonymous substitutions (S342G and I384M).^16^ The G2 allele is a 6-bp deletion (rs71785313/delTTATAA) resulting in the loss of residues N388 and Y389. Under a recessive inheritance model, the presence of homozygous variants (G1/G1 and G2/G2) or compound heterozygosity (G1/G2) significantly increases the risk of CKD development and progression of CKD, characterizing *APOL1* high-risk genotype (APOL1-HR).^7^ In contrast, low risk individuals (APOL1-LR), carrying zero or one allele (G0/G1, G0/G2) present a substantially lower risk, despite emerging evidence suggesting a graded risk compared to G0/G0 individuals.^17^ Although protective factor for trypanosomiasis, these alleles confer risk for kidney disease. *APOL1* risk alleles are associated with increased cellular toxicity, leading to cellular stress, mitochondrial dysfunction, and apoptosis.^18^ Importantly, not all individuals carrying APOL1-HR genotypes develop kidney disease, supporting the “second hit” hypothesis. According to this model, genetic risk alone is insufficient, requiring an additional modifier, biological or environmental, to trigger kidney injury.^19^ Nephropathy associated with viral infections as in HIV and SARS-CoV-2 exposure has been shown to exacerbate *APOL1* mediated toxicity through increased interferon expression, leading to HIV-Associated Nephropathy (HIVAN) and COVID-19-Associated Nephropathy (COVAN) in genetically predisposed individuals.^7^

*APOL1* is responsive to the inflammatory mechanism, particularly interferons (IFN-γ and IFN-α), and under conditions of increased interferon stimulation, *APOL1* expression is upregulated, amplifying cytotoxic effects.^18^ APOL1-HR alters protein's conformation and increases its propensity to pores form in intracellular organelles, inducing ER stress, disrupts ionic homeostasis, activation of pro-apoptotic pathways.^20^ Importantly, mitochondrial dysfunction plays a key role in APOL1 toxicity, as the altered protein disrupts mitochondrial membrane potential, and impairs oxidative phosphorylation increasing the production of reactive oxygen species.^21^ This cascade exacerbates cellular stress and contributes to podocyte injury and kidney damage.

### Biological and clinical framework of APOL1 kidney disease

Clinically, APOL1-HR are associated with earlier onset of CKD, accelerated progression to kidney replacement therapy, and worse renal outcomes.^22^^,^^23^ Hypertension-related CKD is significantly more prevalent in APOL1-HR, and podocyte injury, aggravated by sustained elevated blood pressure, accelerates proteinuria and subsequent renal decline.^24^ Furthermore, systemic inflammation, commonly seen in hypertensive patients, may act as a “second hit” amplifying the *APOL1* mediated cytotoxic effects. The CKD secondary to hypertension is approximately five times more prevalent in Afro-Americans compared to non-Afro-descendant populations.^24^ Among older Afro-Americans (age ≥60-years) in the USA the incidence of CKD-KRT reaches 1500 patients per million population (pmp), nearly three times higher than in the general population, even considering the younger population.^25^ The AASK study demonstrated that APOL1-HR patients have significantly higher risks of CKD progression (HR = 2.21, 95% CI 1.56‒3.14, p < 0.001), doubling creatinine rates and/or a reduction in the estimated glomerular filtration rate (eGFR) at rates 50% of initial value.^23^^,^^24^^,^^26^ According to the cohort analysis, 17% of the individuals had both risk alleles G1 and G2, and of these, 53% developed proteinuria in 6‒8 years.^23^ However, not all *APOL1* risk allele carriers develop hypertensive CKD, highlighting the influence of lifestyle factors, access to treatment, and additional genetic modifiers.

Focal Segmental Glomerulosclerosis (FSGS) represents one of the strongest clinical phenotypes associated with *APOL1* risk variants.^27^ Afro-descendant populations exhibit a four-fold increased risk of developing FSGS with G1 allele is present in 52% and G2 allele in 23%.^27^ The presence of both high-risk alleles conferred a significant risk of developing FSGS, with an Odds Ratio (OR) of 10.5 (95% CI 6.0‒18.4).^27^ These findings were validated in larger cohorts,^28^ which demonstrated the role of the G1/G2 genotype in Afro-Americans with an OR = 17 (95% CI 11‒26) for FSGS, and OR = 29 (95% CI 13‒68) for FSGS associated with HIVAN31. Furthermore, this study also showed an earlier age of disease onset with 70% of patients between 15‒39 years (p = 0.0003), and faster progression to CKD-KRT, with a mean of 5-years for G1/G2 and 13-years for one or none allele (p < 0.01).

HIV-associated nephropathy, the first glomerular collapsing disease described, and it represents a severe renal outcome of HIV infection, strongly associated with APOL1-HR genotypes. Although antiretroviral therapy has markedly reduced its incidence, HIVAN still affects 5%‒10% of HIV-positive individuals.^29^ *APOL1* risk alleles significantly increase the HIVAN susceptibility, with OR ranging from 29 among African Americans to as high as 89 in South African populations.^30^ This outcome is driven by the interplay between the HIV and the *APOL1* G1/G2 risk genotype. HIV infection is a potent inducer of interferons, particularly interferon-γ, which amplifies the expression of *APOL1* in glomerular podocytes.^29^ This overexpression exacerbates cytotoxicity, podocyte injury and apoptosis, leading to severe kidney damage. These phenotypes highlight the clinical relevance of *APOL1* risk variants and underscore the need to investigate their impact in highly admixed populations, such as those found in Brazil.

### Studies on APOL1 kidney disease in the Brazilian population

While APOL1 kidney disease has been extensively investigated in African and African-American populations, studies focusing on the Brazilian population remain limited (Table 1). Available Brazilian studies indicate that individuals carrying two risk alleles are approximately 10 times more likely to develop CKD-KRT and tend to initiate dialysis about 12-years earlier than individuals without genetic risk variants.^27^ These findings underscore the strong influence of APOL1-HR on disease progression and the need for earlier interventions. In Brazil, studies conducted in northeastern and southeastern regions reported similar frequencies of *APOL1* risk alleles among individuals with lupus nephritis and healthy controls, with a low prevalence of individuals carrying both risk alleles.^31^ These results differ from observations in African-American cohorts and suggest regional differences or the contribution of additional genetic and environmental modifiers. In this context, other genetic contributors have been implicated. For example, variants in the *MYH9* gene also have been associated with an increased risk of CKD.^32^ This suggests possible regional differences or the contribution of other genetic and environmental factors, highlighting the complex genetic architecture of kidney disease in admixture populations.

Further investigations in southeastern Brazil have linked the APOL1-HR genotypes to pediatric nephrotic syndrome with accelerated CKD progression even among individuals carrying a single risk allele.^33^^,^^34^ APOL1-HR are associated with pediatric nephrotic syndrome harboring 8% of cases.^33^^,^^35^ underscoring its role in early-onset disease. In Brazilian patients with idiopathic collapsing glomerulopathy, APOL1-HR was strongly associated with African ancestry and severe kidney outcomes with a notable difference in disease progression.^36^^,^^37^ Data show that 58.6% of individuals had causative genetic variants, 80.5% of them with APOL1-HR genotypes, and 19.5% with Mendelian variants.^37^ Ongoing research in Afro-Brazilian quilombola communities provides additional insights.^38^ These studies aim to describe CKD progression and associated risk factors through longitudinal follow-ups, integrating genetic, clinical, and laboratory data to better understand the population's health outcomes. Preliminary findings suggest a significant prevalence of early-stage CKD, emphasizing the need for tailored interventions in these underserved populations.^38^

In Brazil, *APOL1* risk allele frequencies vary across regions, reflecting heterogeneous African ancestry contributions. In northeast Brazil the frequency of risk alleles can reach up to 22% while Afro-Brazilian CKD cohorts from southern Brazil reached G1 15.3% and G2 5.9%.^27^^,^^39^^,^^40^ Risk allele carriers face significantly higher risks of developing lupus nephritis, FSGS, HIVAN, and COVAN, with these risks magnified in individuals carrying both G1 and G2 alleles.^41^, ^42^, ^43^ An extremely important factor to be considered in Brazil, as well as in other tropical countries, is that the diversity of potential pathogens increases the probability of second hit events that trigger CKD in carriers of high-risk genotypes.^37^^,^^44^ These findings collectively illustrate the significant role of *APOL1* variants in shaping kidney disease outcomes in Afro-Brazilians reflecting both local genetic diversity and global trends.

## Discussion

Despite Brazil’s high prevalence and mortality rates associated with CKD, studies investigating the impact of *APOL1* variants in this population remain limited. Existing evidence indicates that Afro-descendants carrying *APOL1* G1 and G2 risk variants have an increased risk of developing CKD, early disease onset, and earlier initiation of kidney replacement therapy. However, Brazil’s unique demographic landscape, marked by its continental extension and a complex mosaic of ethnicities that introduces variability in the distribution and impact of *APOL1* variants across regions. Previous studies have already demonstrated that the presence of *APOL1* variants in the Afro-Brazilian population requires special attention due to the frequency and severity of associated outcomes.^37^ However, the small number of Brazilian studies and their regional concentration highlight substantial knowledge gaps regarding the true burden of APOL1 kidney disease across the country.

Brazil’s highly admixed population represents a unique context for understanding the interaction between genetic ancestry and kidney disease risk. Regional differences in African ancestry contribution are reflected in heterogeneous *APOL1* risk allele frequencies. In different regions the allele frequencies of *APOL1* variants range from 10% to 22%, levels comparable to those observed in African American, Caribbean, and Sub-Saharan African populations.^45^ Similar patterns of high G1 and G2 frequencies have also been reported in Afro-descendant populations in Central America and parts of South America.^25^ Considering the impact of *APOL1* risk alleles on CKD, it becomes evident that more comprehensive studies focused on admixture populations, particularly Afro-Brazilians, are essential for assessing the risk of *APOL1* kidney disease.^46^ The genetic composition of the Brazilian population results from complex historical processes, from the arrival of the first native Americans from Beringia, followed by European colonization, and the transatlantic African slave trade, and subsequent migration waves.^8^^,^^46^ Between the 16th and 19th centuries, Brazil received about half the number of enslaved Black individuals brought to America, with more than four million Africans brought to Brazil, making it the largest recipient of enslaved people.^46^ These historical events shaped the Brazilian population's genetics in a combination of European (60%), African (27%), and Indigenous (13%) components, although these proportions highly vary across regions.^8^^,^^46^ For example, southern Brazil has a higher European contribution (77%), while the northeastern region has a larger Afro-descendant contingent (44%), and the northern region is influenced more by Indigenous ancestry (32%).^8^^,^^46^ Furthermore, the relationship between genetic ancestry and self-identified race in Brazil is not always direct, reflecting the complexity of the miscegenation process and social constructions of race.^8^

All these migration patterns make Brazil an ethnically different country, even when compared to others in Latin America, as well as result in a unique combination of rare and common genetic variants. From a public health perspective, these findings underscore the need for population-specific approaches to CKD risk stratification in Brazil. Individuals with African ancestry, even if they do not identify as Afro-descendants, may carry risk variants such as those in the *APOL1*, and remain outside traditional screening strategies, potentially leading to delayed management. In this context, public health strategies may benefit from improved risk stratification approaches with targeted screening in high-risk regions, increased clinical awareness, and the gradual integration of genetic data into existing epidemiological studies and CKD registries. Previous studies highlighted the importance of including individuals with diverse genetic ancestry to improve clinical practice.^47^ This approach is particularly relevant for highly admixed populations such as Brazil’s, where individuals who are not routinely screened may nevertheless carry high-risk genotypes, increasing vulnerability, underdiagnosis and undertreatment of *APOL1* kidney disease and related coexisting conditions. Limitations of the available literature should be acknowledged. Most Brazilian studies on *APOL1* kidney disease are based on relatively small sample sizes and are geographically concentrated, primarily in Southeast regions, which may limit the generalizability of the findings to the entire Brazilian population. In addition, heterogeneity in study design, ancestry assessment methods, and clinical definitions of CKD further complicates direct comparisons across studies. These limitations highlight the need for larger, multicenter investigations with standardized methodologies and broader regional representation. Ongoing initiatives, including DNA do Brazil Project, as well as population-based studies like ELSA-Brazil, represent valuable resources that could be leveraged for future investigations of *APOL1* risk variants, with detailed clinical, epidemiological, and genetic data.^8^^,^^48^

Therefore, considering the potential impact of APOL1-HR on the health of Afro-descendant populations and the ancestry composition of Brazilians, greater efforts on public health strategies, including education, awareness, prevention, and treatment is needed. Recent advances suggest that targeting interferon pathways may represent as potential therapeutic strategies to modulate APO1 mediated toxicity, supporting the development of tailored precision therapies to improve renal outcomes in this vulnerable population.^2^^,^^31^^,^^33^ Importantly, the emergence of targeted therapies addressing APOL1-mediated toxicity represents a promising avenue for precision medicine. Current treatment strategies for APOL1 kidney disease typically involve antihypertensives, immunosuppressants, and immunomodulators. Ongoing phase 2 and phase 3 clinical trials are evaluating the efficacy and safety of VX-147,^49^ a small molecule that inhibits APOL1 induced cell death in adults with FSGS who carry high-risk *APOL1* variants.^50^ However, the limited representation of Brazilian and other Latin American populations in these trials limits the generalizability of therapeutic advances and reinforces the need for inclusive research efforts.

## Conclusion

*APOL1* kidney disease in Brazil reflects a complex interplay between genetic ancestry, environmental exposures, and social determinants of health. The presence of two high-risk *APOL1* variants are responsible for a substantially increased risk of developing non-diabetic kidney disease in Afro-descendants, leading to earlier age of onset of hemodialysis and worse renal outcomes. In Brazil, a country marked by extensive admixture and regional heterogeneity in African ancestry, evaluating the impact of APOL1-HR is particularly relevant. Accurate interpretation of *APOL1* genotyping becomes important for clinical management, prevention of progression, and risk reduction for Afro-descendants, evidencing health planning strategies for this population. As *APOL1* directed therapies move closer to clinical application, defining who should be tested, when treatment should be initiated, and how conventional therapies interact with *APOL1* kidney disease will be critical, particularly in settings with limited resources.

Considering the discrepancy between the limited number of Brazilian studies and the substantial proportion of the Afro-descendant population in Brazil, there is an urgent need to increase research efforts focused on Afro-Brazilian and admixture populations. Strengthening *APOL1* related research in Brazil is essential to inform evidence-based clinical practice, guide public health policies, and reduce disparities in kidney disease outcomes. Finally, the implementation of *APOL1* testing raises important ethical, social, and policy considerations, including genetic counseling, protection against discrimination, and equitable access to emerging therapies. Addressing these challenges in Brazil will require coordinated efforts to integrate *APOL1* genotyping into nephrology practice, develop national guidelines for genetic counseling, and ensure equitable access to emerging targeted therapies.

## Ethical approval

This study involves a review of published manuscripts available in public databases, according to Brazilian regulation in Resolution CNS-466/2012, there is no need for analysis by the Research Ethics Committee.

### Informed consent to participate

The informed consent is not applicable, as this study does not involve human participants.

## Data availability statement

The data that support the findings of this study are available online at https://doi:10.5281/zenodo.17513953.

## Authors’ contributions

CMBS and BSOF are responsible for conceptualization, methodology, formal analysis, writing the original draft preparation, review, and editing. GCC is responsible for conceptualization, investigation, and review, and PDMMN is responsible for conceptualizing, investigating, and critically revising the work. FVV and FSLV are responsible for conceptualization, methodology, formal analysis, writing the original draft preparation, review and editing, funding acquisition, and supervision.

## Funding acknowledgement

The scholarships needed for this research were funded by the 10.13039/501100007381Instituto Nacional de Genética Médica Populacional (INAGEMP), grant n° CNPq 573993/2008-4, 10.13039/501100004263FAPERGS 17/2551.0000521-0, and 10.13039/501100002322CAPES 88887.136366/2017-00; and Hospital de Clínicas de Porto Alegre (HCPA) ‒ 10.13039/501100006303Fundo de Incentivo à Pesquisa e Eventos (FIPE), grant n° 2020-0474. GCG was the recipient of a 10.13039/501100002322CAPES scholarship grant (grant n° 465549/2014-4). FSLV is the recipient of a 10.13039/501100003593CNPq scholarship grant (grant n° 312960/2021-2).

## Conflicts of interest

The authors Celia M. Barbosa de Souza, Bibiana S. de Oliveira Fam, Giovanna C. Giudicelli, Precil D. M. de Menezes Neves, Francisco V. Veronese, and Fernanda S. Luiz Vianna declare that there are no professional conflicts of interest or financial benefits directly or indirectly arising from the information contained in the manuscript. During the conduct of this study and the preparation of the manuscript, there were no financial, personal, or professional affiliations that could be perceived as influencing the research findings or the content presented.

## Appendix Supplementary materials

Image, application 1

Image, application 2
