# Supplementary material for: An integrative review of APOL1 kidney disease with a focus on the Brazilian population
Source: Clinics (Sao Paulo). 2026 Sep 7;81:101109. doi: 10.1016/j.clinsp.2026.101109 (PMC13579323; doi:10.1016/j.clinsp.2026.101109)
Supplement: Supplementary file 2 [file mmc2.docx]

An integrative review of APOL1 kidney disease with a focus on the Brazilian population

Celia M. Barbosa de Souza ^1,2,3*^, ORCID 0000-0001-6870-1637.

Bibiana S. de Oliveira Fam ^3,4,5*^, ORCID 0000-0002-6654-6415.

Giovanna C. Giudicelli ^3,4^, ORCID 0000-0003-4548-5737.

Precil Diego Miranda de Menezes Neves ^6,7^, ORCID 0000-0003-1089-6763,

Francisco V. Veronese ^1,2^, ORCID 0000-0003-3278-6661.

Fernanda S. Luiz Vianna ^2,3,4,8**^, ORCID 0000-0001-6339-4869.

^1^Nephrology Service, Hospital de Clínicas de Porto Alegre, Federal University of Rio Grande do Sul, Porto Alegre, RS, Brazil; ^2^Graduate Program in Medicine: Medical Sciences, Federal University of Rio Grande do Sul, Porto Alegre, RS, Brazil; ^3^Laboratory of Genomic Medicine, Experimental Research Center, Hospital de Clínicas de Porto Alegre, Porto Alegre, RS, Brazil; ^4^Laboratory of Immunobiology and Immunogenetics, Graduate Program in Genetics and Molecular Biology, Department of Genetics, Institute of Biosciences, Federal University of Rio Grande do Sul, Porto Alegre, RS, Brazil; ^5^Department of Genetics and Evolutionary Biology, Institute of Biosciences, University of São Paulo, São Paulo, SP, Brazil; ^6^Division of Nephrology, Hospital das Clínicas, School of Medicine, University of São Paulo (USP), São Paulo, SP, Brazil; ^7^Center for Nephrology and Dialysis, Oswaldo Cruz German Hospital, São Paulo, SP, Brazil; ^8^INAGEMP, National Institute of Populational Medical Genetics, R. Ramiro Barcelos, 2350 - 21506 - Santa Cecília, Porto Alegre, RS, Brazil. *These authors contributed equally to the study.

**Table S1:** Total of 306 research articles that met all the criteria for original findings on *APOL1* variability in an Afro-descendant population and risk for the development of CKD.

| **Publication Year** | **Location** | **Title** | **Authors** | **Journal/Book** | **PMID** | **Type** |
| --- | --- | --- | --- | --- | --- | --- |
| 2010 | Africa | Missense mutations in the APOL1 gene are highly associated with end stage kidney disease risk previously attributed to the MYH9 gene | Tzur S, Rosset S, Shemer R, Yudkovsky G, Selig S, Tarekegn A, Bekele E, Bradman N, et al. | Hum Genet | 20635188 | Research Article |
| 2010 | North-America | Association of trypanolytic ApoL1 variants with kidney disease in African Americans | Genovese G, Friedman DJ, Ross MD, Lecordier L, Uzureau P, Freedman BI, Bowden DW, Langefeld CD, et al. | Science | 20647424 | Research Article |
| 2010 | North-America | A risk allele for focal segmental glomerulosclerosis in African Americans is located within a region containing APOL1 and MYH9 | Genovese G, Tonna SJ, Knob AU, Appel GB, Katz A, Bernhardy AJ, Needham AW, Lazarus R, et al. | Kidney Int | 20668430 | Research Article |
| 2011 | North-America | The MYH9/APOL1 region and chronic kidney disease in European-Americans | O'Seaghdha CM, Parekh RS, Hwang SJ, Li M, Köttgen A, Coresh J, Yang Q, Fox CS, et al. | Hum Mol Genet | 21429915 | Research Article |
| 2011 | North-America | Genomewide linkage scan for diabetic renal failure and albuminuria: the FIND study | Igo RP Jr, Iyengar SK, Nicholas SB, Goddard KA, Langefeld CD, Hanson RL, Duggirala R, Divers J, et al. | Am J Nephrol | 21454968 | Research Article |
| 2011 | North-America | The APOL1 gene and allograft survival after kidney transplantation | Reeves-Daniel AM, DePalma JA, Bleyer AJ, Rocco MV, Murea M, Adams PL, Langefeld CD, Bowden DW, Hicks PJ, Stratta RJ, et al. | Am J Transplant | 21486385 | Research Article |
| 2011 | North-America | Differential effects of MYH9 and APOL1 risk variants on FRMD3 Association with Diabetic ESRD in African Americans | Freedman BI, Langefeld CD, Lu L, Divers J, Comeau ME, Kopp JB, Winkler CA, Nelson GW, Johnson RC, Palmer ND, et al. | PLoS Genet | 21698141 | Research Article |
| 2011 | North-America | African American living-kidney donors should be screened for APOL1 risk alleles | Cohen DM, Mittalhenkle A, Scott DL, Young CJ, Norman DJ. | Transplantation | 21878839 | Research Article |
| 2011 | North-America | MYH9 and APOL1 are both associated with sickle cell disease nephropathy | Ashley-Koch AE, Okocha EC, Garrett ME, Soldano K, De Castro LM, Jonassaint JC, Orringer EP, Eckman JR, Telen MJ. | Br J Haematol | 21910715 | Research Article |
| 2011 | North-America | Apolipoprotein L1 nephropathy risk variants associate with HDL subfraction concentration in African Americans | Freedman BI, Langefeld CD, Murea M, Ma L, Otvos JD, Turner J, Antinozzi PA, Divers J, Hicks PJ, et al. | Nephrol Dial Transplant | 21931123 | Research Article |
| 2011 | North-America | Absence of APOL1 risk variants protects against HIV-associated nephropathy in the Ethiopian population | Behar DM, Kedem E, Rosset S, Haileselassie Y, Tzur S, Kra-Oz Z, Wasser WG, Shenhar Y, Shahar E, Hassoun G, et al. | Am J Nephrol | 21968148 | Research Article |
| 2011 | North-America | APOL1 localization in normal kidney and nondiabetic kidney disease | Madhavan SM, O'Toole JF, Konieczkowski M, Ganesan S, Bruggeman LA, Sedor JR. | J Am Soc Nephrol | 21997392 | Research Article |
| 2011 | North-America | APOL1 genetic variants in focal segmental glomerulosclerosis and HIV-associated nephropathy | Kopp JB, Nelson GW, Sampath K, Johnson RC, Genovese G, An P, Friedman D, Briggs W, Dart R,et al. | J Am Soc Nephrol | 21997394 | Research Article |
| 2011 | North-America | Population-based risk assessment of APOL1 on renal disease | Friedman DJ, Kozlitina J, Genovese G, Jog P, Pollak MR. | J Am Soc Nephrol | 21997396 | Research Article |
| 2011 | North-America | APOL1 variants increase risk for FSGS and HIVAN but not IgA nephropathy | Papeta N, Kiryluk K, Patel A, Sterken R, Kacak N, Snyder HJ, Imus PH, Mhatre AN, et al. | J Am Soc Nephrol | 21997397 | Research Article |
| 2011 | North-America | Genetic variation in APOL1 associates with younger age at hemodialysis initiation | Kanji Z, Powe CE, Wenger JB, Huang C, Ankers E, Sullivan DA, Collerone G, Powe NR, et al. | J Am Soc Nephrol | 21997398 | Research Article |
| 2012 | Africa | Familial focal segmental glomerulosclerosis (FSGS) in a Nigerian family and exclusion of mutations in NPHS2,WT1 and APOL1 | Anochie IC, Eke FU, Okpere AN. | West Afr J Med | 23468032 | Research Article |
| 2012 | Asia | APOL1 null alleles from a rural village in India do not correlate with glomerulosclerosis | Johnstone DB, Shegokar V, Nihalani D, Rathore YS, Mallik L, Ashish, Zare V, Ikizler HO, Powar R, Holzman LB. | PLoS One | 23300552 | Research Article |
| 2012 | North-America | Polymorphisms in MYH9 are associated with diabetic nephropathy in European Americans | Cooke JN, Bostrom MA, Hicks PJ, Ng MC, Hellwege JN, Comeau ME, Divers J, Langefeld CD, Freedman BI, Bowden DW. | Nephrol Dial Transplant | 21968013 | Research Article |
| 2012 | North-America | Genetic association and gene-gene interaction analyses in African American dialysis patients with nondiabetic nephropathy | Bostrom MA, Kao WH, Li M, Abboud HE, Adler SG, Iyengar SK, Kimmel PL, Hanson RL, Freedman BI, et al.; Family Investigation of Nephropathy and Diabetes (FIND) Research Group. | Am J Kidney Dis | 22119407 | Research Article |
| 2012 | North-America | APOL1 risk variants predict histopathology and progression to ESRD in HIV-related kidney disease | Fine DM, Wasser WG, Estrella MM, Atta MG, Kuperman M, Shemer R, Rajasekaran A, Tzur S, Racusen LC, Skorecki K. | J Am Soc Nephrol | 22135313 | Research Article |
| 2012 | North-America | Role of MYH9 and APOL1 in African and non-African populations with lupus nephritis | Lin CP, Adrianto I, Lessard CJ, Kelly JA, Kaufman KM, Guthridge JM, Freedman BI, Anaya JM, et al. | Genes Immun | 22189356 | Research Article |
| 2012 | North-America | APOL1 allelic variants are associated with lower age of dialysis initiation and thereby increased dialysis vintage in African and Hispanic Americans with non-diabetic end-stage kidney disease | Tzur S, Rosset S, Skorecki K, Wasser WG. | Nephrol Dial Transplant | 22357707 | Research Article |
| 2012 | North-America | HIV-associated nephropathy patients with and without apolipoprotein L1 gene variants have similar clinical and pathological characteristics | Atta MG, Estrella MM, Kuperman M, Foy MC, Fine DM, Racusen LC, Lucas GM, Nelson GW, Warner AC, Winkler CA, Kopp JB. | Kidney Int | 22495294 | Research Article |
| 2012 | North-America | Association of APOL1 variants with mild kidney disease in the first-degree relatives of African American patients with non-diabetic end-stage renal disease | Freedman BI, Langefeld CD, Turner J, Núñez M, High KP, Spainhour M, Hicks PJ, Bowden DW, Reeves-Daniel AM, Murea M, Rocco MV, Divers J. | Kidney Int | 22695330 | Research Article |
| 2012 | North-America | Collapsing glomerulopathy associated lupus in a black female with homozygous APOL1 mutation | Kofman T, Narjoz C, Raimbourg Q, Loriot MA, Karras A, Roland M, Hill G, Jacquot C, Nochy D, Thervet E. | Lupus | 22952321 | Research Article |
| 2012 | North-America | Multiple loci associated with renal function in African Americans | Shriner D, Herbert A, Doumatey AP, Zhou J, Huang H, Erdos MR, Chen G, Gerry NP, Christman MF, Adeyemo A, Rotimi CN. | PLoS One | 23028791 | Research Article |
| 2013 | Africa | Genetic variation in APOL1 and MYH9 genes is associated with chronic kidney disease among Nigerians | Tayo BO, Kramer H, Salako BL, Gottesman O, McKenzie CA, Ogunniyi A, Bottinger EP, Cooper RS. | Int Urol Nephrol | 22956460 | Research Article |
| 2013 | Africa | Identifying Darwinian selection acting on different human APOL1 variants among diverse African populations | Ko WY, Rajan P, Gomez F, Scheinfeldt L, An P, Winkler CA, Froment A, Nyambo TB, Omar SA, Wambebe C, Ranciaro A, Hirbo JB, Tishkoff SA. | Am J Hum Genet | 23768513 | Research Article |
| 2013 | Africa | High population frequencies of APOL1 risk variants are associated with increased prevalence of non-diabetic chronic kidney disease in the Igbo people from south-eastern Nigeria | Ulasi II, Tzur S, Wasser WG, Shemer R, Kruzel E, Feigin E, Ijoma CK, Onodugo OD, Okoye JU, Arodiwe EB, Ifebunandu NA, Chukwuka CJ, Onyedum CC, Ijoma UN, Nna E, Onuigbo M, Rosset S, Skorecki K. | Nephron Clin Pract | 23860441 | Research Article |
| 2013 | Africa | Targeted exome sequencing integrated with clinicopathological information reveals novel and rare mutations in atypical, suspected and unknown cases of Alport syndrome or proteinuria | Chatterjee R, Hoffman M, Cliften P, Seshan S, Liapis H, Jain S. | PLoS One | 24130771 | Research Article |
| 2013 | Asia | Epistatic role of the MYH9/APOL1 region on familial hematuria genes | Voskarides K, Demosthenous P, Papazachariou L, Arsali M, Athanasiou Y, Zavros M, Stylianou K, Xydakis D, et al. | PLoS One | 23516419 | Research Article |
| 2013 | North-America | Apolipoprotein L1 gene variants associate with hypertension-attributed nephropathy and the rate of kidney function decline in African Americans | Lipkowitz MS, Freedman BI, Langefeld CD, Comeau ME, Bowden DW, Kao WH, Astor BC, Bottinger EP, et al. | Kidney Int | 22832513 | Research Article |
| 2013 | North-America | Hypertension-misattributed kidney disease in African Americans | Skorecki KL, Wasser WG. | Kidney Int | 23271482 | Research Article |
| 2013 | North-America | APOL1 variants and kidney disease in people of recent African ancestry | Genovese G, Friedman DJ, Pollak MR. | Nat Rev Nephrol | 23438974 | Research Article |
| 2013 | North-America | Apolipoprotein L1 risk variants associate with systemic lupus erythematosus-associated collapsing glomerulopathy | Larsen CP, Beggs ML, Saeed M, Walker PD. | J Am Soc Nephrol | 23520206 | Research Article |
| 2013 | North-America | JC polyoma virus interacts with APOL1 in African Americans with nondiabetic nephropathy | Divers J, Núñez M, High KP, Murea M, Rocco MV, Ma L, Bowden DW, Hicks PJ, et al. | Kidney Int | 23677244 | Research Article |
| 2013 | North-America | APOL1 variants associate with increased risk of CKD among African Americans | Foster MC, Coresh J, Fornage M, Astor BC, Grams M, Franceschini N, Boerwinkle E, Parekh RS, Kao WH. | J Am Soc Nephrol | 23766536 | Research Article |
| 2013 | North-America | APOL1 risk variants, race, and progression of chronic kidney disease | Parsa A, Kao WH, Xie D, Astor BC, Li M, Hsu CY, Feldman HI, Parekh RS, Kusek JW, et al; AASK Study Investigators; CRIC Study Investigators. | N Engl J Med | 24206458 | Research Article |
| 2013 | North-America | Rapid detection and quantification of apolipoprotein L1 genetic variants and total levels in plasma by ultra-performance liquid chromatography/tandem mass spectrometry | Zhou H, Hoek M, Yi P, Rohm RJ, Mahsut A, Brown P, Saunders J, Chmielowski RA, et al. | Rapid Commun Mass Spectrom | 24591025 | Research Article |
| 2014 | Central-America | APOL1 polymorphisms and development of CKD in an identical twin donor and recipient pair | Kofman T, Audard V, Narjoz C, Gribouval O, Matignon M, Leibler C, Desvaux D, Lang P, Grimbert P. | Am J Kidney Dis | 24518129 | Research Article |
| 2014 | North-America | Gene-gene interactions in APOL1-associated nephropathy | Divers J, Palmer ND, Lu L, Langefeld CD, Rocco MV, Hicks PJ, Murea M, Ma L, Bowden DW, Freedman BI. | Nephrol Dial Transplant | 24157943 | Research Article |
| 2014 | North-America | Plasma apolipoprotein L1 levels do not correlate with CKD | Bruggeman LA, O'Toole JF, Ross MD, Madhavan SM, Smurzynski M, Wu K, Bosch RJ, Gupta S, Pollak MR, Sedor JR, Kalayjian RC. | J Am Soc Nephrol | 24231663 | Research Article |
| 2014 | North-America | Generalization of associations of kidney-related genetic loci to American Indians | Franceschini N, Haack K, Almasy L, Laston S, Lee ET, Best LG, Fabsitz RR, MacCluer JW, Howard BV, Umans JG, Cole SA. | Clin J Am Soc Nephrol | 24311711 | Research Article |
| 2014 | North-America | Increased burden of cardiovascular disease in carriers of APOL1 genetic variants | Ito K, Bick AG, Flannick J, Friedman DJ, Genovese G, Parfenov MG, Depalma SR, Gupta N, Gabriel SB, et al. | Circ Res | 24379297 | Research Article |
| 2014 | North-America | Analysis of coding variants identified from exome sequencing resources for association with diabetic and non-diabetic nephropathy in African Americans | Bailey JNC, Palmer ND, Ng MCY, Bonomo JA, Hicks PJ, Hester JM, Langefeld CD, Freedman BI, Bowden DW. | Hum Genet | 24385048 | Research Article |
| 2014 | North-America | Podocyte-associated gene mutation screening in a heterogeneous cohort of patients with sporadic focal segmental glomerulosclerosis | Laurin LP, Lu M, Mottl AK, Blyth ER, Poulton CJ, Weck KE. | Nephrol Dial Transplant | 24500309 | Research Article |
| 2014 | North-America | End-stage renal disease in African Americans with lupus nephritis is associated with APOL1 | Freedman BI, Langefeld CD, Andringa KK, Croker JA, Williams AH, Garner NE, Birmingham DJ, Hebert LA, et al.; Lupus Nephritis–End‐Stage Renal Disease Consortium. | Arthritis Rheumatol | 24504811 | Research Article |
| 2014 | North-America | Evaluation of candidate nephropathy susceptibility genes in a genome-wide association study of African American diabetic kidney disease | Palmer ND, Ng MC, Hicks PJ, Mudgal P, Langefeld CD, Freedman BI, Bowden DW. | PLoS One | 24551085 | Research Article |
| 2014 | North-America | Explaining the racial difference in AKI incidence | Grams ME, Matsushita K, Sang Y, Estrella MM, Foster MC, Tin A, Kao WH, Coresh J. | J Am Soc Nephrol | 24722442 | Research Article |
| 2014 | North-America | APOL1 risk variants enhance podocyte necrosis through compromising lysosomal membrane permeability | Lan X, Jhaveri A, Cheng K, Wen H, Saleem MA, Mathieson PW, Mikulak J, Aviram S, Malhotra A, Skorecki K, Singhal PC. | Am J Physiol Renal Physiol | 24899058 | Research Article |
| 2014 | South-America | MYH9 and APOL1 gene polymorphisms and the risk of CKD in patients with lupus nephritis from an admixture population | Colares VS, Titan SM, Pereira Ada C, Malafronte P, Cardena MM, Santos S, Santos PC, Fridman C, Barros RT, Woronik V. | PLoS One | 24658608 | Research Article |
| 2015 | Africa | APOL1 genetic variants, chronic kidney diseases and hypertension in mixed ancestry South Africans | Matsha TE, Kengne AP, Masconi KL, Yako YY, Erasmus RT. | BMC Genet | 26112018 | Research Article |
| 2015 | Africa | Human Heredity and Health (H3) in Africa Kidney Disease Research Network: A Focus on Methods in Sub-Saharan Africa | Osafo C, Raji YR, Burke D, Tayo BO, Tiffin N, Moxey-Mims MM, Rasooly RS, Kimmel PL, Ojo A, Adu D, Parekh RS; H3Africa Kidney Disease Research Network Investigators as members of The H3Africa Consortium. | Clin J Am Soc Nephrol | 26138261 | Research Article |
| 2015 | North-America | Localization of APOL1 protein and mRNA in the human kidney: nondiseased tissue, primary cells, and immortalized cell lines | Ma L, Shelness GS, Snipes JA, Murea M, Antinozzi PA, Cheng D, Saleem MA, Satchell SC, Banas B, Mathieson PW, Kretzler M, Hemal AK, Rudel LL, Petrovic S, Weckerle A, Pollak MR, Ross MD, Parks JS, Freedman BI. | J Am Soc Nephrol | 25012173 | Research Article |
| 2015 | North-America | Apolipoprotein L1 gene variants associate with prevalent kidney but not prevalent cardiovascular disease in the Systolic Blood Pressure Intervention Trial | Langefeld CD, Divers J, Pajewski NM, Hawfield AT, Reboussin DM, Bild DE, Kaysen GA, Kimmel PL, Raj DS, Ricardo AC, Wright JT Jr, Sedor JR, Rocco MV, Freedman BI; Systolic Blood Pressure Intervention Trial (SPRINT). | Kidney Int | 25029429 | Research Article |
| 2015 | North-America | APOL1 associations with nephropathy, atherosclerosis, and all-cause mortality in African Americans with type 2 diabetes | Freedman BI, Langefeld CD, Lu L, Palmer ND, Smith SC, Bagwell BM, Hicks PJ, Xu J, Wagenknecht LE, Raffield LM, Register TC, Carr JJ, Bowden DW, Divers J. | Kidney Int | 25054777 | Research Article |
| 2015 | North-America | Histopathologic findings associated with APOL1 risk variants in chronic kidney disease | Larsen CP, Beggs ML, Saeed M, Ambruzs JM, Cossey LN, Messias NC, Walker PD, Freedman BI. | Mod Pathol | 25081748 | Research Article |
| 2015 | North-America | Innate immunity pathways regulate the nephropathy gene Apolipoprotein L1 | Nichols B, Jog P, Lee JH, Blackler D, Wilmot M, D'Agati V, Markowitz G, Kopp JB, Alper SL, Pollak MR, Friedman DJ. | Kidney Int | 25100047 | Research Article |
| 2015 | North-America | The association between APOL1 risk alleles and longitudinal kidney function differs by HIV viral suppression status | Estrella MM, Li M, Tin A, Abraham AG, Shlipak MG, Penugonda S, Hussain SK, Palella FJ Jr, Wolinsky SM, Martinson JJ, Parekh RS, Kao WH. | Clin Infect Dis | 25281610 | Research Article |
| 2015 | North-America | Effect of Genetic African Ancestry on eGFR and Kidney Disease | Udler MS, Nadkarni GN, Belbin G, Lotay V, Wyatt C, Gottesman O, Bottinger EP, Kenny EE, Peter I. | J Am Soc Nephrol | 25349204 | Research Article |
| 2015 | North-America | Estimating time to ESRD using kidney failure risk equations: results from the African American Study of Kidney Disease and Hypertension (AASK) | Grams ME, Li L, Greene TH, Tin A, Sang Y, Kao WH, Lipkowitz MS, Wright JT, Chang AR, Astor BC, Appel LJ. | Am J Kidney Dis | 25441435 | Research Article |
| 2015 | North-America | Clinical phenotype of APOL1 nephropathy in young relatives of patients with end-stage renal disease | Anyaegbu EI, Shaw AS, Hruska KA, Jain S. | Pediatr Nephrol | 25530085 | Research Article |
| 2015 | North-America | Clinical Features and Histology of Apolipoprotein L1-Associated Nephropathy in the FSGS Clinical Trial | Kopp JB, Winkler CA, Zhao X, Radeva MK, Gassman JJ, D'Agati VD, Nast CC, Wei C, Reiser J, Guay-Woodford LM, Pollak MR, Hildebrandt F, Moxey-Mims M, Gipson DS, Trachtman H, Friedman AL, Kaskel FJ; FSGS-CT Study Consortium. | J Am Soc Nephrol | 25573908 | Research Article |
| 2015 | North-America | APOL1 Risk Variants Are Strongly Associated with HIV-Associated Nephropathy in Black South Africans | Kasembeli AN, Duarte R, Ramsay M, Mosiane P, Dickens C, Dix-Peek T, Limou S, Sezgin E, Nelson GW, Fogo AB, Goetsch S, Kopp JB, Winkler CA, Naicker S. | J Am Soc Nephrol | 25788523 | Research Article |
| 2015 | North-America | Vascular smooth muscle cells contribute to APOL1-induced podocyte injury in HIV milieu | Lan X, Wen H, Saleem MA, Mikulak J, Malhotra A, Skorecki K, Singhal PC. | Exp Mol Pathol | 25796344 | Research Article |
| 2015 | North-America | Apolipoprotein L1 gene variants in deceased organ donors are associated with renal allograft failure | Freedman BI, Julian BA, Pastan SO, Israni AK, Schladt D, Gautreaux MD, Hauptfeld V, Bray RA, Gebel HM, Kirk AD, Gaston RS, Rogers J, Farney AC, Orlando G, Stratta RJ, Mohan S, Ma L, Langefeld CD, Hicks PJ, Palmer ND, Adams PL, Palanisamy A, Reeves-Daniel AM, Divers J. | Am J Transplant | 25809272 | Research Article |
| 2015 | North-America | Deceased donor multidrug resistance protein 1 and caveolin 1 gene variants may influence allograft survival in kidney transplantation | Ma J, Divers J, Palmer ND, Julian BA, Israni AK, Schladt D, Pastan SO, Chattrabhuti K, Gautreaux MD, Hauptfeld V, Bray RA, et al. | Kidney Int | 25853335 | Research Article |
| 2015 | North-America | Apolipoprotein L1, income and early kidney damage | Tamrat R, Peralta CA, Tajuddin SM, Evans MK, Zonderman AB, Crews DC. | BMC Nephrol | 25884165 | Research Article |
| 2015 | North-America | Hemostatic Factors, APOL1 Risk Variants, and the Risk of ESRD in the Atherosclerosis Risk in Communities Study | Tin A, Grams ME, Maruthur NM, Astor BC, Couper D, Mosley TH, Fornage M, Parekh RS, Coresh J, Kao WH. | Clin J Am Soc Nephrol | 25887069 | Research Article |
| 2015 | North-America | APOL1 Genotype and Glomerular and Tubular Kidney Injury in Women With HIV | Jotwani V, Shlipak MG, Scherzer R, Parekh RS, Kao WH, Bennett M, Cohen MH, Nowicki M, Sharma A, Young M, Tien PC, Parikh CR, Estrella MM. | Am J Kidney Dis | 25921719 | Research Article |
| 2015 | North-America | Copy Number Variation at the APOL1 Locus | Ruchi R, Genovese G, Lee J, Charoonratana VT, Bernhardy AJ, Alper SL, Kopp JB, Thadhani R, Friedman DJ, Pollak MR. | PLoS One | 25933006 | Research Article |
| 2015 | North-America | Sequencing rare and common APOL1 coding variants to determine kidney disease risk | Limou S, Nelson GW, Lecordier L, An P, O'hUigin CS, David VA, Binns-Roemer EA, Guiblet WM, Oleksyk TK, Pays E, Kopp JB, Winkler CA. | Kidney Int | 25993319 | Research Article |
| 2015 | North-America | APOL1 G1 genotype modifies the association between HDLC and kidney function in African Americans | Bentley AR, Divers J, Shriner D, Doumatey AP, Gutiérrez OM, Adeyemo AA, Freedman BI, Rotimi CN. | BMC Genomics | 26025194 | Research Article |
| 2015 | North-America | APOL1 Risk Alleles Are Associated with Exaggerated Age-Related Changes in Glomerular Number and Volume in African-American Adults: An Autopsy Study | Hoy WE, Hughson MD, Kopp JB, Mott SA, Bertram JF, Winkler CA. | J Am Soc Nephrol | 26038529 | Research Article |
| 2015 | North-America | Testing for High-Risk APOL1 Alleles in Potential Living Kidney Donors | Riella LV, Sheridan AM. | Am J Kidney Dis | 26049628 | Research Article |
| 2015 | North-America | Protein domains of APOL1 and its risk variants | Lan X, Wen H, Lederman R, Malhotra A, Mikulak J, Popik W, Skorecki K, Singhal PC. | Exp Mol Pathol | 26091559 | Research Article |
| 2015 | North-America | In vivo Modeling Implicates APOL1 in Nephropathy: Evidence for Dominant Negative Effects and Epistasis under Anemic Stress | Anderson BR, Howell DN, Soldano K, Garrett ME, Katsanis N, Telen MJ, Davis EE, Ashley-Koch AE. | PLoS Genet | 26147622 | Research Article |
| 2015 | North-America | Re-Sequencing of the APOL1-APOL4 and MYH9 Gene Regions in African Americans Does Not Identify Additional Risks for CKD Progression | Hawkins GA, Friedman DJ, Lu L, McWilliams DR, Chou JW, Sajuthi S, Divers J, Parekh RS, Li M, Genovese G, Pollack MR, Hicks PJ, Bowden DW, Ma L, Freedman BI, Langefeld CD. | Am J Nephrol | 26343748 | Research Article |
| 2015 | North-America | Examination of Potential Modifiers of the Association of APOL1 Alleles with CKD Progression | Chen TK, Choi MJ, Kao WH, Astor BC, Scialla JJ, Appel LJ, Li L, Lipkowitz MS, Wolf M, Parekh RS, Winkler CA, Estrella MM, Crews DC. | Clin J Am Soc Nephrol | 26430087 | Research Article |
| 2016 | Africa | Brief Report: APOL1 Renal Risk Variants Are Associated With Chronic Kidney Disease in Children and Youth With Perinatal HIV Infection | Purswani MU, Patel K, Winkler CA, Spector SA, Hazra R, Seage GR 3rd, Mofenson L, Karalius B, Scott GB, Van Dyke RB, Kopp JB; Pediatric HIVAIDS Cohort Study. | J Acquir Immune Defic Syndr | 27035887 | Research Article |
| 2016 | Europe | Impact of APOL1 polymorphism and IL-1β priming in the entry and persistence of HIV-1 in human podocytes | Mikulak J, Oriolo F, Portale F, Tentorio P, Lan X, Saleem MA, Skorecki K, Singhal PC, Mavilio D. | Retrovirology | 27599995 | Research Article |
| 2016 | North-America | Integrative Genomics Identifies Novel Associations with APOL1 Risk Genotypes in Black NEPTUNE Subjects | Sampson MG, Robertson CC, Martini S, Mariani LH, Lemley KV, Gillies CE, Otto EA, Kopp JB, Randolph A, Vega-Warner V, Eichinger F, Nair V, Gipson DS, Cattran DC, Johnstone DB, O'Toole JF, Bagnasco SM, Song PX, Barisoni L, Troost JP, Kretzler M, Sedor JR; Nephrotic Syndrome Study Network. | J Am Soc Nephrol | 26150607 | Research Article |
| 2016 | North-America | APOL1 nephropathy risk variants are associated with altered high-density lipoprotein profiles in African Americans | Gutiérrez OM, Judd SE, Irvin MR, Zhi D, Limdi N, Palmer ND, Rich SS, Sale MM, Freedman BI. | Nephrol Dial Transplant | 26152403 | Research Article |
| 2016 | North-America | APOL1 Genotype and Race Differences in Incident Albuminuria and Renal Function Decline | Peralta CA, Bibbins-Domingo K, Vittinghoff E, Lin F, Fornage M, Kopp JB, Winkler CA. | J Am Soc Nephrol | 26180129 | Research Article |
| 2016 | North-America | Characterization of circulating APOL1 protein complexes in African Americans | Weckerle A, Snipes JA, Cheng D, Gebre AK, Reisz JA, Murea M, Shelness GS, Hawkins GA, Furdui CM, Freedman BI, Parks JS, Ma L. | J Lipid Res | 26586272 | Research Article |
| 2016 | North-America | APOL1 Genotype, Kidney and Cardiovascular Disease, and Death in Older Adults | Mukamal KJ, Tremaglio J, Friedman DJ, Ix JH, Kuller LH, Tracy RP, Pollak MR. | Arterioscler Thromb Vasc Biol | 26634651 | Research Article |
| 2016 | North-America | Association of APOL1 Genotype with Renal Histology among Black HIV-Positive Patients Undergoing Kidney Biopsy | Atta MG, Estrella MM, Skorecki KL, Kopp JB, Winkler CA, Wasser WG, Shemer R, Racusen LC, Kuperman M, Foy MC, Lucas GM, Fine DM. | Clin J Am Soc Nephrol | 26668025 | Research Article |
| 2016 | North-America | APOL1 kidney disease risk variants cause cytotoxicity by depleting cellular potassium and inducing stress-activated protein kinases | Olabisi OA, Zhang JY, VerPlank L, Zahler N, DiBartolo S 3rd, Heneghan JF, Schlöndorff JS, Suh JH, Yan P, Alper SL, Friedman DJ, Pollak MR. | Proc Natl Acad Sci U S A | 26699492 | Research Article |
| 2016 | North-America | Determining the effects and challenges of incorporating genetic testing into primary care management of hypertensive patients with African ancestry | Horowitz CR, Abul-Husn NS, Ellis S, Ramos MA, Negron R, Suprun M, Zinberg RE, Sabin T, Hauser D, Calman N, Bagiella E, Bottinger EP. | Contemp Clin Trials | 26747051 | Research Article |
| 2016 | North-America | Analytical Validation of a Personalized Medicine APOL1 Genotyping Assay for Nondiabetic Chronic Kidney Disease Risk Assessment | Zhang J, Fedick A, Wasserman S, Zhao G, Edelmann L, Bottinger EP, Kornreich R, Scott SA. | J Mol Diagn | 26773863 | Research Article |
| 2016 | North-America | APOL1 Variants: From Parasites to Kidney Function to Cardiovascular Disease | Reiner AP, Susztak K. | Arterioscler Thromb Vasc Biol | 26819463 | Research Article |
| 2016 | North-America | A role for genetic susceptibility in sporadic focal segmental glomerulosclerosis | Yu H, Artomov M, Brähler S, Stander MC, Shamsan G, Sampson MG, White JM, Kretzler M, Miner JH, Jain S, Winkler CA, Mitra RD, Kopp JB, Daly MJ, Shaw AS. | J Clin Invest | 26901816 | Research Article |
| 2016 | North-America | Combined Effects of GSTM1 Null Allele and APOL1 Renal Risk Alleles in CKD Progression in the African American Study of Kidney Disease and Hypertension Trial | Bodonyi-Kovacs G, Ma JZ, Chang J, Lipkowitz MS, Kopp JB, Winkler CA, Le TH. | J Am Soc Nephrol | 26940095 | Research Article |
| 2016 | North-America | Structural characterization of the C-terminal coiled-coil domains of wild-type and kidney disease-associated mutants of apolipoprotein L1 | Sharma AK, Friedman DJ, Pollak MR, Alper SL. | FEBS J | 26945671 | Research Article |
| 2016 | North-America | Race, APOL1 Risk, and eGFR Decline in the General Population | Grams ME, Rebholz CM, Chen Y, Rawlings AM, Estrella MM, Selvin E, Appel LJ, Tin A, Coresh J. | J Am Soc Nephrol | 26966015 | Research Article |
| 2016 | North-America | Deceased-Donor Apolipoprotein L1 Renal-Risk Variants Have Minimal Effects on Liver Transplant Outcomes | Dorr CR, Freedman BI, Hicks PJ, Brown WM, Russell GB, Julian BA, Pastan SO, Gautreaux MD, Muthusamy A, Chinnakotla S, Hauptfeld V, Bray RA, Kirk AD, Divers J, Israni AK. | PLoS One | 27054572 | Research Article |
| 2016 | North-America | APOL1 renal-risk genotypes associate with longer hemodialysis survival in prevalent nondiabetic African American patients with end-stage renal disease | Ma L, Langefeld CD, Comeau ME, Bonomo JA, Rocco MV, Burkart JM, Divers J, Palmer ND, Hicks PJ, Bowden DW, Lea JP, Krisher JO, Clay MJ, Freedman BI. | Kidney Int | 27157696 | Research Article |
| 2016 | North-America | Patterns of Kidney Function Decline Associated with APOL1 Genotypes: Results from AASK | Tin A, Grams ME, Estrella M, Lipkowitz M, Greene TH, Kao WH, Li L, Appel LJ. | Clin J Am Soc Nephrol | 27230965 | Research Article |
| 2016 | North-America | APOL1 renal-risk variants associate with reduced cerebral white matter lesion volume and increased gray matter volume | Freedman BI, Gadegbeku CA, Bryan RN, Palmer ND, Hicks PJ, Ma L, Rocco MV, Smith SC, Xu J, Whitlow CT, Wagner BC, Langefeld CD, Hawfield AT, Bates JT, Lerner AJ, Raj DS, Sadaghiani MS, Toto RD, Wright JT Jr, Bowden DW, Williamson JD, Sink KM, Maldjian JA, Pajewski NM, Divers J. | Kidney Int | 27342958 | Research Article |
| 2016 | North-America | Association of kidney structure-related gene variants with type 2 diabetes-attributed end-stage kidney disease in African Americans | Guan M, Ma J, Keaton JM, Dimitrov L, Mudgal P, Stromberg M, Bonomo JA, Hicks PJ, Freedman BI, Bowden DW, Ng MC. | Hum Genet | 27461219 | Research Article |
| 2016 | North-America | From man to fish: What can Zebrafish tell us about ApoL1 nephropathy? | Olabisi O, Al-Romaih K, Henderson J, Tomar R, Drummond I, MacRae C, Pollak M. | Clin Nephrol | 27509583 | Research Article |
| 2016 | North-America | Collapsing glomerulopathy in a young woman with APOL1 risk alleles following acute parvovirus B19 infection: a case report investigation | Besse W, Mansour S, Jatwani K, Nast CC, Brewster UC. | BMC Nephrol | 27600725 | Research Article |
| 2017 | Africa | Clinical and genetic predictors of renal dysfunctions in sickle cell anaemia in Cameroon | Geard A, Pule GD, Chetcha Chemegni B, Ngo Bitoungui VJ, Kengne AP, Chimusa ER, Wonkam A. | Br J Haematol | 28466968 | Research Article |
| 2017 | Africa | APOL1 renal risk variants have contrasting resistance and susceptibility associations with African trypanosomiasis | Cooper A, Ilboudo H, Alibu VP, Ravel S, Enyaru J, Weir W, Noyes H, Capewell P, Camara M, Milet J, Jamonneau V, Camara O, Matovu E, Bucheton B, MacLeod A. | Elife | 28537557 | Research Article |
| 2017 | Africa | MicroRNAs in the miR-17 and miR-15 families are downregulated in chronic kidney disease with hypertension | Nandakumar P, Tin A, Grove ML, Ma J, Boerwinkle E, Coresh J, Chakravarti A. | PLoS One | 28771472 | Research Article |
| 2017 | Africa | Association Between APOL1 Genotypes and Risk of Cardiovascular Disease in MESA (Multi-Ethnic Study of Atherosclerosis) | Chen TK, Katz R, Estrella MM, Gutierrez OM, Kramer H, Post WS, Shlipak MG, Wassel CL, Peralta CA. | J Am Heart Assoc | 29269352 | Research Article |
| 2017 | Asia | Intracellular APOL1 Risk Variants Cause Cytotoxicity Accompanied by Energy Depletion | Granado D, Müller D, Krausel V, Kruzel-Davila E, Schuberth C, Eschborn M, Wedlich-Söldner R, Skorecki K, Pavenstädt H, Michgehl U, Weide T. | J Am Soc Nephrol | 28696248 | Research Article |
| 2017 | Asia | Does copy number variation of APOL1 gene affect the susceptibility to focal segmental glomerulosclerosis? | Peng T, Li G, Zhong X, Wang L. | Ren Fail | 28494221 | Research Article |
| 2017 | North-America | APOL1-associated glomerular disease among African-American children: a collaboration of the Chronic Kidney Disease in Children (CKiD) and Nephrotic Syndrome Study Network (NEPTUNE) cohorts | Ng DK, Robertson CC, Woroniecki RP, Limou S, Gillies CE, Reidy KJ, Winkler CA, et al. | Nephrol Dial Transplant | 27190333 | Research Article |
| 2017 | North-America | An investigation of APOL1 risk genotypes and preterm birth in African American population cohorts | Robertson CC, Gillies CE, Putler RKB, Ng D, Reidy KJ, Crawford B, Sampson MG. | Nephrol Dial Transplant | 27638911 | Research Article |
| 2017 | North-America | African Ancestry-Specific Alleles and Kidney Disease Risk in Hispanics/Latinos | Kramer HJ, Stilp AM, Laurie CC, Reiner AP, Lash J, Daviglus ML, Rosas SE, et al. | J Am Soc Nephrol | 27650483 | Research Article |
| 2017 | North-America | APOL1, α-thalassemia, and BCL11A variants as a genetic risk profile for progression of chronic kidney disease in sickle cell anemia | Saraf SL, Shah BN, Zhang X, Han J, Tayo BO, Abbasi T, Ostrower A, Guzman E, et al. | Haematologica | 27658436 | Research Article |
| 2017 | North-America | APOL1 Renal-Risk Variants Induce Mitochondrial Dysfunction | Ma L, Chou JW, Snipes JA, Bharadwaj MS, Craddock AL, Cheng D, Weckerle A, Petrovic S, Hicks PJ, Hemal AK, Hawkins GA, Miller LD, Molina AJ, Langefeld CD, Murea M, Parks JS, Freedman BI. | J Am Soc Nephrol | 27821631 | Research Article |
| 2017 | North-America | APOL1-G1 in Nephrocytes Induces Hypertrophy and Accelerates Cell Death | Fu Y, Zhu JY, Richman A, Zhang Y, Xie X, Das JR, Li J, Ray PE, Han Z. | J Am Soc Nephrol | 27864430 | Research Article |
| 2017 | North-America | APOL1-Mediated Cell Injury Involves Disruption of Conserved Trafficking Processes | Kruzel-Davila E, Shemer R, Ofir A, Bavli-Kertselli I, Darlyuk-Saadon I, Oren-Giladi P, Wasser WG, et al. | J Am Soc Nephrol | 27864431 | Research Article |
| 2017 | North-America | Strict blood pressure control associates with decreased mortality risk by APOL1 genotype | Ku E, Lipkowitz MS, Appel LJ, Parsa A, Gassman J, Glidden DV, Smogorzewski M, Hsu CY. | Kidney Int | 27927600 | Research Article |
| 2017 | North-America | Most ApoL1 Is Secreted by the Liver | Shukha K, Mueller JL, Chung RT, Curry MP, Friedman DJ, Pollak MR, Berg AH. | J Am Soc Nephrol | 27932478 | Research Article |
| 2017 | North-America | APOL1 variants may induce HIV-associated nephropathy during HIV primary infection | De Laroche M, Desbuissons G, Rouvier P, Barin F, Deray G, Caumes E, Katlama C, Tubiana R, Isnard Bagnis C. | J Antimicrob Chemother | 28100445 | Research Article |
| 2017 | North-America | Transgenic expression of human APOL1 risk variants in podocytes induces kidney disease in mice | Beckerman P, Bi-Karchin J, Park AS, Qiu C, Dummer PD, Soomro I, Boustany-Kari CM, Pullen SS, et al. | Nat Med | 28218918 | Research Article |
| 2017 | North-America | Race, Genomics and Chronic Disease: What Patients with African Ancestry Have to Say | Horowitz CR, Ferryman K, Negron R, Sabin T, Rodriguez M, Zinberg RF, Böttinger E, Robinson M. | J Health Care Poor Underserved | 28238999 | Research Article |
| 2017 | North-America | Sickle Cell Trait and the Risk of ESRD in Blacks | Naik RP, Irvin MR, Judd S, Gutiérrez OM, Zakai NA, Derebail VK, Peralta C, Lewis MR, Zhi D, Arnett D, McClellan W, Wilson JG, Reiner AP, Kopp JB, Winkler CA, Cushman M. | J Am Soc Nephrol | 28280138 | Research Article |
| 2017 | North-America | APOL1 genetic variants are not associated with longitudinal blood pressure in young black adults | Chen TK, Estrella MM, Vittinghoff E, Lin F, Gutierrez OM, Kramer H, Lewis CE, Kopp JB, Allen NB, Winkler CA, Bibbins-Domingo KB, Peralta CA. | Kidney Int | 28545715 | Research Article |
| 2017 | North-America | APOL1 Risk Variants and Cardiovascular Disease: Results From the AASK (African American Study of Kidney Disease and Hypertension) | Chen TK, Appel LJ, Grams ME, Tin A, Choi MJ, Lipkowitz MS, Winkler CA, Estrella MM. | Arterioscler Thromb Vasc Biol | 28572159 | Research Article |
| 2017 | North-America | A tripartite complex of suPAR, APOL1 risk variants and α(v)β(3) integrin on podocytes mediates chronic kidney disease | Hayek SS, Koh KH, Grams ME, Wei C, Ko YA, Li J, Samelko B, Lee H, Dande RR, Lee HW, et al. | Nat Med | 28650456 | Research Article |
| 2017 | North-America | Roles of APOL1 G1 and G2 variants in sickle cell disease patients: kidney is the main target | Kormann R, Jannot AS, Narjoz C, Ribeil JA, Manceau S, Delville M, Joste V, Prié D, Pouchot J, Thervet E, Courbebaisse M, Arlet JB. | Br J Haematol | 28699644 | Research Article |
| 2017 | North-America | APOL1 risk alleles among individuals with CKD in Northern Tanzania: A pilot study | Stanifer JW, Karia F, Maro V, Kilonzo K, Qin X, Patel UD, Hauser ER. | PLoS One | 28732083 | Research Article |
| 2017 | North-America | APOL1 Risk Variants, Incident Proteinuria, and Subsequent eGFR Decline in Blacks with Hypertension-Attributed CKD | Chen TK, Tin A, Peralta CA, Appel LJ, Choi MJ, Lipkowitz MS, Winkler CA, Estrella MM. | Clin J Am Soc Nephrol | 29051146 | Research Article |
| 2018 | Africa | No evidence for association between APOL1 kidney disease risk alleles and Human African Trypanosomiasis in two Ugandan populations | Kimuda MP, Noyes H, Mulindwa J, Enyaru J, Alibu VP, Sidibe I, Mumba Ngoyi D, Hertz-Fowler C, et al. | PLoS Negl Trop Dis | 29470556 | Research Article |
| 2018 | Africa | The sickle cell trait and end stage renal disease in Salvador, Brazil | Alladagbin DJ, Fernandes PN, Tavares MB, Brito JT, Oliveira GGS, Silva LK, Khouri NA, Oliveira MB, Amorim T, Matos CM, Ribeiro GS, Lopes AA, Gonçalves MS, Dos-Santos WLC. | PLoS One | 30557319 | Research Article |
| 2018 | North-America | Apolipoprotein L1 and Chronic Kidney Disease Risk in Young Potential Living Kidney Donors | Locke JE, Sawinski D, Reed RD, Shelton B, MacLennan PA, Kumar V, Mehta S, Mannon RB, Gaston R, Julian BA, Carr JJ, Terry JG, Kilgore M, Massie AB, Segev DL, Lewis CE. | Ann Surg | 28187045 | Research Article |
| 2018 | North-America | Obesity and synergistic risk factors for chronic kidney disease in African American adults: the Jackson Heart Study | Olivo RE, Davenport CA, Diamantidis CJ, Bhavsar NA, Tyson CC, Hall R, Bidulescu A, Young B, Mwasongwe SE, Pendergast J, Boulware LE, Scialla JJ. | Nephrol Dial Transplant | 28992354 | Research Article |
| 2018 | North-America | APOL1 nephropathy risk variants do not associate with subclinical atherosclerosis or left ventricular mass in middle-aged black adults | Gutiérrez OM, Limou S, Lin F, Peralta CA, Kramer HJ, Carr JJ, Bibbins-Domingo K, Winkler CA, Lewis CE, Kopp JB. | Kidney Int | 29042080 | Research Article |
| 2018 | North-America | ApoL1 Overexpression Drives Variant-Independent Cytotoxicity | O'Toole JF, Schilling W, Kunze D, Madhavan SM, Konieczkowski M, Gu Y, Luo L, Wu Z, Bruggeman LA, Sedor JR. | J Am Soc Nephrol | 29180397 | Research Article |
| 2018 | North-America | Clinical and genetic factors are associated with pain and hospitalisation rates in sickle cell anaemia in Cameroon | Wonkam A, Mnika K, Ngo Bitoungui VJ, Chetcha Chemegni B, Chimusa ER, Dandara C, Kengne AP. | Br J Haematol | 29205277 | Research Article |
| 2018 | North-America | HLA-DQA1 and APOL1 as Risk Loci for Childhood-Onset Steroid-Sensitive and Steroid-Resistant Nephrotic Syndrome | Adeyemo A, Esezobor C, Solarin A, Abeyagunawardena A, Kari JA, El Desoky S, Greenbaum LA, et al. | Am J Kidney Dis | 29277510 | Research Article |
| 2018 | North-America | APOL1 Genotype and Renal Function of Black Living Donors | Doshi MD, Ortigosa-Goggins M, Garg AX, Li L, Poggio ED, Winkler CA, Kopp JB. | J Am Soc Nephrol | 29339549 | Research Article |
| 2018 | North-America | ApoL1 renal risk variants induce aberrant THP-1 monocyte differentiation and increase eicosanoid production via enhanced expression of cyclooxygenase-2 | Lee H, Roshanravan H, Wang Y, Okamoto K, Ryu J, Shrivastav S, Qu P, Kopp JB. | Am J Physiol Renal Physiol | 29357411 | Research Article |
| 2018 | North-America | JC polyoma viruria associates with protection from chronic kidney disease independently from apolipoprotein L1 genotype in African Americans | Freedman BI, Kistler AL, Skewes-Cox P, Ganem D, Spainhour M, Turner J, Divers J, et al. | Nephrol Dial Transplant | 29420808 | Research Article |
| 2018 | North-America | UBD modifies APOL1-induced kidney disease risk | Zhang JY, Wang M, Tian L, Genovese G, Yan P, Wilson JG, Thadhani R, Mottl AK, Appel GB, Bick AG, Sampson MG, Alper SL, Friedman DJ, Pollak MR. | Proc Natl Acad Sci U S A | 29531077 | Research Article |
| 2018 | North-America | Plasma biomarkers are associated with renal outcomes in individuals with APOL1 risk variants | Nadkarni GN, Chauhan K, Verghese DA, Parikh CR, Do R, Horowitz CR, Bottinger EP, Coca SG. | Kidney Int | 29685497 | Research Article |
| 2018 | North-America | Serum 6-Bromotryptophan Levels Identified as a Risk Factor for CKD Progression | Tin A, Nadkarni G, Evans AM, Winkler CA, Bottinger E, Rebholz CM, Sarnak MJ, Inker LA, et al. | J Am Soc Nephrol | 29777021 | Research Article |
| 2018 | North-America | Blocking the 5' splice site of exon 4 by a morpholino oligomer triggers APOL1 protein isoform switch | Cheatham AM, Davis SE, Khatua AK, Popik W. | Sci Rep | 29880816 | Research Article |
| 2018 | North-America | Genome-wide association studies suggest that APOL1-environment interactions more likely trigger kidney disease in African Americans with nondiabetic nephropathy than strong APOL1-second gene interactions | Langefeld CD, Comeau ME, Ng MCY, Guan M, Dimitrov L, Mudgal P, Spainhour MH, Julian BA, et al. | Kidney Int | 29885931 | Research Article |
| 2018 | North-America | APOL1 Nephropathy Risk Variants and Incident Cardiovascular Disease Events in Community-Dwelling Black Adults | Gutiérrez OM, Irvin MR, Chaudhary NS, Cushman M, Zakai NA, David VA, Limou S, Pamir N, et al. | Circ Genom Precis Med | 29899045 | Research Article |
| 2018 | North-America | APOL1 risk variants cause podocytes injury through enhancing endoplasmic reticulum stress | Wen H, Kumar V, Lan X, Shoshtari SSM, Eng JM, Zhou X, Wang F, Wang H, Skorecki K, Xing G, Wu G, Luo H, Malhotra A, Singhal PC. | Biosci Rep | 29967295 | Research Article |
| 2018 | North-America | Association of APOL1 With Heart Failure With Preserved Ejection Fraction in Postmenopausal African American Women | Franceschini N, Kopp JB, Barac A, Martin LW, Li Y, Qian H, Reiner AP, Pollak M, et al. | JAMA Cardiol | 29971324 | Research Article |
| 2018 | South-America | Worldwide Frequencies of APOL1 Renal Risk Variants | Nadkarni GN, Gignoux CR, Sorokin EP, Daya M, Rahman R, Barnes KC, Wassel CL, Kenny EE. | N Engl J Med | 30586505 | Research Article |
| 2019 | Africa | Low prevalence of apolipoprotein L1 gene variants in Black South Africans with hypertension-attributed chronic kidney disease | Nqebelele NU, Dickens C, Dix-Peek T, Duarte R, Naicker S. | Clin Nephrol | 30369398 | Research Article |
| 2019 | Africa | Optimal management of HIV- positive adults at risk for kidney disease in Nigeria (Renal Risk Reduction "R3" Trial): protocol and study design | Aliyu MH, Wudil UJ, Ingles DJ, Shepherd BE, Gong W, Musa BM, Muhammad H, Sani MU, et al. | Trials | 31182139 | Research Article |
| 2019 | Africa | Association of APOL1 renal disease risk alleles with Trypanosoma brucei rhodesiense infection outcomes in the northern part of Malawi | Kamoto K, Noyes H, Nambala P, Senga E, Musaya J, Kumwenda B, Bucheton B, et al. | PLoS Negl Trop Dis | 31412021 | Research Article |
| 2019 | Africa | The podocin V260E mutation predicts steroid resistant nephrotic syndrome in black South African children with focal segmental glomerulosclerosis | Govender MA, Fabian J, Gottlich E, Levy C, Moonsamy G, Maher H, Winkler CA, Ramsay M. | Commun Biol | 31754646 | Research Article |
| 2019 | North-America | APOL1 risk genotype in European steroid-resistant nephrotic syndrome and/or focal segmental glomerulosclerosis patients of different African ancestries | Gribouval O, Boyer O, Knebelmann B, Karras A, Dantal J, Fourrage C, Alibeu O, Hogan J, Dossier C, Tête MJ, Antignac C, Servais A. | Nephrol Dial Transplant | 29992269 | Research Article |
| 2019 | North-America | Recruitment of APOL1 kidney disease risk variants to lipid droplets attenuates cell toxicity | Chun J, Zhang JY, Wilkins MS, Subramanian B, Riella C, Magraner JM, Alper SL, Friedman DJ, Pollak MR. | Proc Natl Acad Sci U S A | 30733285 | Research Article |
| 2019 | North-America | Impact of APOL1 Genetic Variants on HIV-1 Infection and Disease Progression | An P, Kirk GD, Limou S, Binns-Roemer E, Kopp JB, Winkler CA. | Front Immunol | 30733721 | Research Article |
| 2019 | North-America | Survival Advantage of African American Dialysis Patients with End-Stage Renal Disease Causes Related to APOL1 | Lertdumrongluk P, Streja E, Rhee CM, Moradi H, Chang Y, Reddy U, Tantisattamo E, Kalantar-Zadeh K, Kopp JB. | Cardiorenal Med | 30995638 | Research Article |
| 2019 | North-America | APOL1 renal risk variants promote cholesterol accumulation in tissues and cultured macrophages from APOL1 transgenic mice | Ryu JH, Ge M, Merscher S, Rosenberg AZ, Desante M, Roshanravan H, Okamoto K, Shin MK, Hoek M, Fornoni A, Kopp JB. | PLoS One | 30998685 | Research Article |
| 2019 | North-America | Association of APOL1 Risk Alleles With Cardiovascular Disease in Blacks in the Million Veteran Program | Bick AG, Akwo E, Robinson-Cohen C, Lee K, Lynch J, Assimes TL, DuVall S, Edwards T, et al. | Circulation | 31337231 | Research Article |
| 2019 | North-America | Sickle cell trait, estimated glomerular filtration rate, and risk of adverse outcomes in chronic kidney disease | Sood R, Surapaneni A, Luo S, Appel LJ, Winkler C, Grams ME, Naik RP. | Am J Hematol | 31342549 | Research Article |
| 2019 | North-America | Apolipoprotein L1 Testing in African Americans: Involving the Community in Policy Discussions | Young BA, Blacksher E, Cavanaugh KL, Freedman BI, Fullerton SM, Kopp JB, Umeukeje EM, West KM, Wilson JG, Burke W; APOL1 Stakeholders Project. | Am J Nephrol | 31480040 | Research Article |
| 2019 | North-America | APOL1 Kidney Risk Variants Induce Cell Death via Mitochondrial Translocation and Opening of the Mitochondrial Permeability Transition Pore | Shah SS, Lannon H, Dias L, Zhang JY, Alper SL, Pollak MR, Friedman DJ. | J Am Soc Nephrol | 31558683 | Research Article |
| 2019 | North-America | Apolipoprotein L1 (APOL1) risk variant toxicity depends on the haplotype background | Lannon H, Shah SS, Dias L, Blackler D, Alper SL, Pollak MR, Friedman DJ. | Kidney Int | 31611067 | Research Article |
| 2019 | North-America | APOL1 Nephropathy Risk Alleles and Risk of Sepsis in Blacks | Chaudhary NS, Moore JX, Zakai NA, Judd SE, Naik RP, Limou S, Cushman M, Lange LA, et al. | Clin J Am Soc Nephrol | 31704668 | Research Article |
| 2019 | North-America | A Focus Group Study on African American Living Donors' Treatment Preferences, Sociocultural Factors, and Health Beliefs About Apolipoprotein L1 Genetic Testing | Gordon EJ, Amórtegui D, Blancas I, Wicklund C, Friedewald J, Sharp RR. | Prog Transplant | 31146624 | Research Article |
| 2019 | South-America | APOL1-Associated Kidney Disease in Brazil | Riella C, Siemens TA, Wang M, Campos RP, Moraes TP, Riella LV, Friedman DJ, Riella MC, Pollak MR. | Kidney Int Rep | 31317114 | Research Article |
| 2020 | Africa | Association of Genetic Polymorphisms of TGF-β1, HMOX1, and APOL1 With CKD in Nigerian Patients With and Without HIV | Ekrikpo UE, Mnika K, Effa EE, Ajayi SO, Okwuonu C, Waziri B, Bello A, Dandara C, Kengne AP, Wonkam A, Okpechi I. | Am J Kidney Dis | 32354559 | Research Article |
| 2020 | Africa | APOL1 Genetic Variants Are Associated with Serum-Oxidized Low-Density Lipoprotein Levels and Subclinical Atherosclerosis in South African CKD Patients | Hassan MO, Duarte R, Dickens C, Dix-Peek T, Naidoo S, Vachiat A, Grinter S, Manga P, Naicker S. | Nephron | 32526749 | Research Article |
| 2020 | Africa | Design and methods of the prevalence and pharmacogenomics of tenofovir nephrotoxicity in HIV-positive adults in south-western Nigeria study | Hassan MO, Duarte R, Mabayoje VO, Dickens C, Lasisi AO, Naicker S. | BMC Nephrol | 33066744 | Research Article |
| 2020 | Europe | Malaria, Collapsing Glomerulopathy, and Focal and Segmental Glomerulosclerosis | Amoura A, Moktefi A, Halfon M, Karras A, Rafat C, Gibier JB, Gleeson PJ, Servais A, et al. | Clin J Am Soc Nephrol | 32444394 | Research Article |
| 2020 | North-America | APOL1 Nephropathy Risk Alleles and Mortality in African American Adults: A Cohort Study | Gutiérrez OM, Irvin MR, Zakai NA, Naik RP, Chaudhary NS, Estrella MM, Limou S, Judd SE, Cushman M, Kopp JB, Winkler CA. | Am J Kidney Dis | 31563468 | Research Article |
| 2020 | North-America | APOL1-Associated Collapsing Focal Segmental Glomerulosclerosis in a Patient With Stimulator of Interferon Genes (STING)-Associated Vasculopathy With Onset in Infancy (SAVI) | Abid Q, Best Rocha A, Larsen CP, Schulert G, Marsh R, Yasin S, Patty-Resk C, Valentini RP, Adams M, Baracco R. | Am J Kidney Dis | 31601430 | Research Article |
| 2020 | North-America | Frequency of ClinVar Pathogenic Variants in Chronic Kidney Disease Patients Surveyed for Return of Research Results at a Cleveland Public Hospital | Crawford DC, Lin J, Cooke Bailey JN, Kinzy T, Sedor JR, O'Toole JF, Bush WS. | Pac Symp Biocomput | 31797629 | Research Article |
| 2020 | North-America | Variation of ApoL1 Testing Practices for Living Kidney Donors | McIntosh T, Mohan S, Sawinski D, Iltis A, DuBois JM. | Prog Transplant | 31838940 | Research Article |
| 2020 | North-America | APOL1 Genotyping in Potential African American Living Kidney Donors: Utility and Cost-Effectiveness | Gudsoorkar P, Anand M, Abu Jawdeh BG. | Am J Nephrol | 31940609 | Research Article |
| 2020 | North-America | Genome-wide association study for time to failure of kidney transplants from African American deceased donors | Divers J, Ma L, Brown WM, Palmer ND, Choi Y, Israni AK, Pastan SO, Julian BA, Gaston RS, Hicks PJ, Reeves-Daniel AM, Freedman BI. | Clin Transplant | 32080893 | Research Article |
| 2020 | North-America | Initial experience from a renal genetics clinic demonstrates a distinct role in patient management | Thomas CP, Freese ME, Ounda A, Jetton JG, Holida M, Noureddine L, Smith RJ. | Genet Med | 32203225 | Research Article |
| 2020 | North-America | Phenome-wide association analysis suggests the APOL1 linked disease spectrum primarily drives kidney-specific pathways | Bajaj A, Ihegword A, Qiu C, Small AM, Wei WQ, Bastarache L, Feng Q, Kember RL, Risman M, Bloom RD, Birtwell DL, Williams H, Shaffer CM, Chen J, Center RG, Denny JC, Rader DJ, Stein CM, Damrauer SM, Susztak K. | Kidney Int | 32247630 | Research Article |
| 2020 | North-America | Apolipoprotein L1 (APOL1) Coding Variants Are Associated With Creatinine Rise After Cardiac Surgery | Privratsky JR, Li YJ, Haynes C, Podgoreanu M, Mathew J, Shah SH, Stafford-Smith M. | J Cardiothorac Vasc Anesth | 32434723 | Research Article |
| 2020 | North-America | AKI and Collapsing Glomerulopathy Associated with COVID-19 and APOL 1 High-Risk Genotype | Wu H, Larsen CP, Hernandez-Arroyo CF, Mohamed MMB, Caza T, Sharshir M, Chughtai A, Xie L, Gimenez JM, Sandow TA, Lusco MA, Yang H, Acheampong E, Rosales IA, Colvin RB, Fogo AB, Velez JCQ. | J Am Soc Nephrol | 32561682 | Research Article |
| 2020 | North-America | Profiling APOL1 Nephropathy Risk Variants in Genome-Edited Kidney Organoids with Single-Cell Transcriptomics | Liu E, Radmanesh B, Chung BH, Donnan MD, Yi D, Dadi A, Smith KD, Himmelfarb J, Li M, Freedman BS, Lin J. | Kidney360 | 32656538 | Research Article |
| 2020 | North-America | Association of APOL1 Genotypes With Measures of Microvascular and Endothelial Function, and Blood Pressure in MESA | Chen TK, Katz R, Estrella MM, Post WS, Kramer H, Rotter JI, Tayo B, Mychaleckyj JC, Wassel CL, Peralta CA. | J Am Heart Assoc | 32851884 | Research Article |
| 2020 | North-America | An Acidic Environment Induces APOL1-Associated Mitochondrial Fragmentation | Li D, Snipes JA, Murea M, Molina AJA, Divers J, Freedman BI, Ma L, Petrovic S. | Am J Nephrol | 32866949 | Research Article |
| 2020 | North-America | Beyond Panel-Based Testing: Exome Analysis Increases Sensitivity for Diagnosis of Genetic Kidney Disease | Wilson PC, Love-Gregory L, Corliss M, McNulty S, Heusel JW, Gaut JP. | Kidney360 | 35372954 | Research Article |
| 2020 | South-America | Effect of a Single Apolipoprotein L1 Gene Nephropathy Variant on the Risk of Advanced Lupus Nephritis in Brazilians | Vajgel G, Lima SC, Santana DJS, Oliveira CBL, Costa DMN, Hicks PJ, Cavalcante MAGM, Langefeld CD, Valente LM, Crovella S, Kirsztajn GM, Freedman BI, Sandrin-Garcia P. | J Rheumatol | 31732553 | Research Article |
| 2021 | Africa | Apolipoprotein L1 risk genotypes in Ghanaian patients with systemic lupus erythematosus: a prospective cohort study | Blazer A, Dey ID, Nwaukoni J, Reynolds M, Ankrah F, Algasas H, Ahmed T, Divers J. | Lupus Sci Med | 33461980 | Research Article |
| 2021 | Africa | Apolipoprotein-1 risk variants and associated kidney phenotypes in an adult HIV cohort in Nigeria | Wudil UJ, Aliyu MH, Prigmore HL, Ingles DJ, Ahonkhai AA, Musa BM, Muhammad H, et al. | Kidney Int | 33901548 | Research Article |
| 2021 | Africa | Novel Human Podocyte Cell Model Carrying G2/G2 APOL1 High-Risk Genotype | Ekulu PM, Adebayo OC, Decuypere JP, Bellucci L, Elmonem MA, Nkoy AB, Mekahli D, Bussolati B, van den Heuvel LP, Arcolino FO, Levtchenko EN. | Cells | 34440683 | Research Article |
| 2021 | North-America | Race, APOL1 Risk Variants, and Clinical Outcomes among Older Adults: The ARIC Study | Chen TK, Coresh J, Daya N, Ballew SH, Tin A, Crews DC, Grams ME. | J Am Geriatr Soc | 32894582 | Research Article |
| 2021 | North-America | Joint Associations of Maternal-Fetal APOL1 Genotypes and Maternal Country of Origin With Preeclampsia Risk | Hong X, Rosenberg AZ, Zhang B, Binns-Roemer E, David V, Lv Y, Hjorten RC, Reidy KJ, Chen TK, Wang G, Ji Y, Simpson CL, Davis RL, Kopp JB, Wang X, Winkler CA. | Am J Kidney Dis | 33359152 | Research Article |
| 2021 | North-America | Kidney-disease-associated variants of Apolipoprotein L1 show gain of function in cation channel activity | Bruno J, Edwards JC. | J Biol Chem | 33380423 | Research Article |
| 2021 | North-America | Biomarkers of Immune Activation and Incident Kidney Failure With Replacement Therapy: Findings From the African American Study of Kidney Disease and Hypertension | Chen TK, Estrella MM, Appel LJ, Coresh J, Luo S, Reiser J, Obeid W, Parikh CR, Grams ME. | Am J Kidney Dis | 33388403 | Research Article |
| 2021 | North-America | Podocyte density is reduced in kidney allografts with high-risk APOL1 genotypes at transplantation | Chen DP, Zaky ZS, Schold JD, Herlitz LC, El-Rifai R, Drawz PE, Bruggeman LA, Barisoni L, Hogan SL, Hu Y, O'Toole JF, Poggio ED, Sedor JR. | Clin Transplant | 33511679 | Research Article |
| 2021 | North-America | APOL1 risk variants affect podocyte lipid homeostasis and energy production in focal segmental glomerulosclerosis | Ge M, Molina J, Ducasa GM, Mallela SK, Varona Santos J, Mitrofanova A, Kim JJ, Liu X,et al. | Hum Mol Genet | 33517446 | Research Article |
| 2021 | North-America | APOL1 genotype-associated morphologic changes among patients with focal segmental glomerulosclerosis | Zee J, McNulty MT, Hodgin JB, Zhdanova O, Hingorani S, Jefferson JA, Gibson KL, Trachtman H, et al. | Pediatr Nephrol | 33646395 | Research Article |
| 2021 | North-America | APOL1 variant alleles associate with reduced risk for opportunistic infections in HIV infection | An P, Sezgin E, Kirk GD, Duggal P, Binns-Roemer E, Nelson G, Limou S, Van Natta ML, Jabs DA, Estrella M, Kopp JB, Winkler CA. | Commun Biol | 33674766 | Research Article |
| 2021 | North-America | APOL1 Genetic Variants Are Associated With Increased Risk of Coronary Atherosclerotic Plaque Rupture in the Black Population | Cornelissen A, Fuller DT, Fernandez R, Zhao X, Kutys R, Binns-Roemer E, Delsante M, Sakamoto A, et al. | Arterioscler Thromb Vasc Biol | 34039022 | Research Article |
| 2021 | North-America | SNPs at 3'UTR of APOL1 and miR-6741-3p target sites associated with kidney diseases more susceptible to SARS-COV-2 infection: in silco and in vitro studies | Safdar M, Khan MS, Karim AY, Omar SA, Smail SW, Saeed M, Zaheer S, Ali M, Ahmad B, Tasleem M, Junejo Y. | Mamm Genome | 34089082 | Research Article |
| 2021 | North-America | A multi-center retrospective cohort study defines the spectrum of kidney pathology in Coronavirus 2019 Disease (COVID-19) | May RM, Cassol C, Hannoudi A, Larsen CP, Lerma EV, Haun RS, Braga JR, Hassen SI,et al. | Kidney Int | 34352311 | Research Article |
| 2021 | North-America | Glomerular Disease in Temporal Association with SARS-CoV-2 Vaccination: A Series of 29 Cases | Caza TN, Cassol CA, Messias N, Hannoudi A, Haun RS, Walker PD, May RM, Seipp RM, et al. | Kidney360 | 35372991 | Research Article |
| 2021 | North-America | APOL1 Risk Variants and Acute Kidney Injury in Black Americans with COVID-19 | Larsen CP, Wickman TJ, Braga JR, Matute-Trochez LA, Hasty AE, Buckner LR, Arthur JM, Haun RS, Velez JCQ. | Clin J Am Soc Nephrol | 36630401 | Research Article |
| 2021 | South-America | APOL1 in an ethnically diverse pediatric population with nephrotic syndrome: implications in focal segmental glomerulosclerosis and other diagnoses | Watanabe A, Guaragna MS, Belangero VMS, Casimiro FMS, Pesquero JB, de Santis Feltran L, Palma LMP, Varela P, et al,. | Pediatr Nephrol | 33585978 | Research Article |
| 2022 | Africa | Clinical and genetic factors are associated with kidney complications in African children with sickle cell anaemia | Adebayo OC, Betukumesu DK, Nkoy AB, Adesoji OM, Ekulu PM, Van den Heuvel LP, Levtchenko EN, Labarque V. | Br J Haematol | 34545573 | Research Article |
| 2022 | Africa | Apolipoprotein L1 High-Risk Genotypes and Albuminuria in Sub-Saharan African Populations | Brandenburg JT, Govender MA, Winkler CA, Boua PR, Agongo G, Fabian J, Ramsay M. | Clin J Am Soc Nephrol | 35577564 | Research Article |
| 2022 | Africa | Obesity-related glomerulopathy in the presence of APOL1 risk alleles | Valdez Imbert R, Hti Lar Seng NS, Stokes MB, Jim B. | BMJ Case Rep | 35985743 | Research Article |
| 2022 | Africa | Molecular Dynamic Simulation Reveals Structure Differences in APOL1 Variants and Implication in Pathogenesis of Chronic Kidney Disease | Mayanja R, Kintu C, Diabate O, Soremekun O, Oluwagbemi OO, Wele M, Kalyesubula R, Jjingo D, Chikowore T, Fatumo S. | Genes (Basel) | 36011371 | Research Article |
| 2022 | Africa | High risk APOL1 genotypes and kidney disease among treatment naïve HIV patients at Kano, Nigeria | Abdu A, Duarte R, Dickens C, Dix-Peek T, Bala SM, Ademola B, Naicker S. | PLoS One | 36227935 | Research Article |
| 2022 | Africa | Chronic kidney disease (CKD) and associated risk in rural South Africa: a population-based cohort study | Fabian J, Gondwe M, Mayindi N, Chipungu S, Khoza B, Gaylard P, Wade AN, et al. | Wellcome Open Res | 36457874 | Research Article |
| 2022 | Africa | Urine-Based Detection of Biomarkers Indicative of Chronic Kidney Disease in a Patient Cohort from Ghana | Wruck W, Boima V, Erichsen L, Thimm C, Koranteng T, Kwakyi E, Antwi S, Adu D, Adjaye J. | J Pers Med | 36675700 | Research Article |
| 2022 | Europe | Distinct APOL1 functions in trypanosomes and kidney podocytes | Pays E. | Trends Parasitol | 34887168 | Research Article |
| 2022 | Europe | Apolipoprotein L1 variability is associated with increased risk of renal failure in the Czech population | Hubacek JA, Hruba P, Adamkova V, Pokorna E, Viklicky O. | Gene | 35085711 | Research Article |
| 2022 | North-America | Whole Genome Sequence Analysis of the Plasma Proteome in Black Adults Provides Novel Insights Into Cardiovascular Disease | Katz DH, Tahir UA, Bick AG, Pampana A, Ngo D, Benson MD, Yu Z, Robbins JM, Chen ZZ, et al. | Circulation | 34814699 | Research Article |
| 2022 | North-America | Stakeholder Perspectives on Returning Nonactionable Apolipoprotein L1 (APOL1) Genetic Results to African American Research Participants | West KM, Cavanaugh KL, Blacksher E, Fullerton SM, Umeukeje EM, Young B, Burke W. | J Empir Res Hum Res Ethics | 34870514 | Research Article |
| 2022 | North-America | A glomerular transcriptomic landscape of apolipoprotein L1 in Black patients with focal segmental glomerulosclerosis | McNulty MT, Fermin D, Eichinger F, Jang D, Kretzler M, Burtt NP, Pollak MR, Flannick J, Weins A, Friedman DJ; Nephrotic Syndrome Study Network (NEPTUNE), Sampson MG. | Kidney Int | 34929253 | Research Article |
| 2022 | North-America | Employment status at transplant influences ethnic disparities in outcomes after deceased donor kidney transplantation | Divers J, Mohan S, Brown WM, Pastan SO, Israni AK, Gaston RS, Bray R, Islam S, Sakhovskaya NV, Mena-Gutierrez AM, Reeves-Daniel AM, Julian BA, Freedman BI. | BMC Nephrol | 34979953 | Research Article |
| 2022 | North-America | APOL1 Risk Variants, Acute Kidney Injury, and Death in Participants With African Ancestry Hospitalized With COVID-19 From the Million Veteran Program | Hung AM, Shah SC, Bick AG, Yu Z, Chen HC, Hunt CM, Wendt F, Wilson O, et al. | JAMA Intern Med | 35089317 | Research Article |
| 2022 | North-America | APOL1 Risk Variants Associated with Serum Albumin in a Population-Based Cohort Study | Chaudhary NS, Tiwari HK, Hidalgo BA, Limdi NA, Reynolds RJ, Cushman M, Zakai NA, Lange L, Judd SE, Winkler CA, Kopp JB, Gutiérrez OM, Irvin MR. | Am J Nephrol | 35100591 | Research Article |
| 2022 | North-America | Effects of Testing and Disclosing Ancestry-Specific Genetic Risk for Kidney Failure on Patients and Health Care Professionals: A Randomized Clinical Trial | Nadkarni GN, Fei K, Ramos MA, Hauser D, Bagiella E, Ellis SB, Sanderson S, Scott SA, Sabin T, Madden E, Cooper R, Pollak M, Calman N, Bottinger EP, Horowitz CR. | JAMA Netw Open | 35244702 | Research Article |
| 2022 | North-America | Associations between APOL1 genetic variants and blood pressure in African American mothers and children from a U.S. pregnancy cohort: Modification by air pollution exposures | Ni Y, Simpson CL, Davis RL, Szpiro AA, Karr CJ, Kovesdy CP, Hjorten RC, Tylavsky FA, Bush NR, LeWinn KZ, Winkler CA, Kopp JB, Obi Y. | Environ Res | 35358541 | Research Article |
| 2022 | North-America | JAK inhibitor blocks COVID-19 cytokine-induced JAK/STAT/APOL1 signaling in glomerular cells and podocytopathy in human kidney organoids | Nystrom SE, Li G, Datta S, Soldano KL, Silas D, Weins A, Hall G, Thomas DB, Olabisi OA. | JCI Insight | 35472001 | Research Article |
| 2022 | North-America | APOL1 Kidney Risk Variants and Proteomics | Chen TK, Surapaneni AL, Arking DE, Ballantyne CM, Boerwinkle E, Chen J, Coresh J, Köttgen A, Susztak K, Tin A, Yu B, Grams ME. | Clin J Am Soc Nephrol | 35474272 | Research Article |
| 2022 | North-America | Genome-wide polygenic score to predict chronic kidney disease across ancestries | Khan A, Turchin MC, Patki A, Srinivasasainagendra V, Shang N, Nadukuru R, Jones AC, Malolepsza E, et al. | Nat Med | 35710995 | Research Article |
| 2022 | North-America | Rates of Cardiovascular Disease and CKD Progression in Young Adults with CKD across Racial and Ethnic Groups | Kula AJ, Prince DK, Limonte CP, Young BA, Bansal N. | Kidney360 | 36128489 | Research Article |
| 2022 | North-America | SMOC2 gene interacts with APOL1 in the development of end-stage kidney disease: A genome-wide association study | Chaudhary NS, Armstrong ND, Hidalgo BA, Gutiérrez OM, Hellwege JN, Limdi NA, Reynolds RJ, Judd SE, Nadkarni GN, Lange L, Winkler CA, Kopp JB, Arnett DK, Tiwari HK, Irvin MR. | Front Med (Lausanne) | 36250097 | Research Article |
| 2022 | North-America | A Case of COVID-Associated Nephropathy (COVAN) | Craig T, Ansari M, Foster P, Abdelgadir Y, Abdelghani A, Jha P. | Cureus | 36465729 | Research Article |
| 2022 | North-America | APOL1 Genotype, Proteinuria, and the Risk of Kidney Failure: A Secondary Analysis of the AASK (African American Study of Kidney Disease and Hypertension) and CRIC (Chronic Renal Insufficiency Cohort) Studies | Nguyen A, Suen SC, Lin E. | Kidney Med | 36479469 | Research Article |
| 2022 | North-America | Transcriptomic Analysis of Human Podocytes In Vitro: Effects of Differentiation and APOL1 Genotype | Yoshida T, Latt KZ, Rosenberg AZ, Shrivastav S, Heymann J, Halushka MK, Winkler CA, Kopp JB. | Kidney Int Rep | 36644347 | Research Article |
| 2022 | South-America | APOL1 genotyping via buccal mucosa cell samples to establish risk of kidney disease | Alladagbin DJ, da Silva CGR, Silva LK, Dos-Santos WL, de Sá Oliveira GG. | BMC Nephrol | 36217118 | Research Article |
| 2023 | Africa | Rationale and Design of the Diet, CKD, and Apolipoprotein L1 Study in Low-Income and Middle-Income Countries | Ilori TO, Solarin A, Manmak M, Raji YR, Braimoh R, Kwakyi E, Umeizudike T, Ajepe T,et al. | Kidney Int Rep | 37069986 | Research Article |
| 2023 | Central-America | Urinary Metabolomic Profile of Youth at Risk of Chronic Kidney Disease in Nicaragua | Hall SM, Raines NH, Ramirez-Rubio O, Amador JJ, López-Pilarte D, O'Callaghan-Gordo C, Gil-Redondo R, Embade N, Millet O, Peng X, Vences S, Keogh SA, Delgado IS, Friedman DJ, Brooks DR, Leibler JH. | Kidney360 | 37068179 | Research Article |
| 2023 | Europe | Genetic testing in focal segmental glomerulosclerosis: in whom and when? | Tato AM, Carrera N, García-Murias M, Shabaka A, Ávila A, Mora Mora MT, Rabasco C, Soto K, et al. | Clin Kidney J | 37915894 | Research Article |
| 2023 | North-America | Phenotypes of APOL1 High-Risk Status Subjects | Kopp JB, Yoshida T. | J Am Soc Nephrol | 37126670 | Research Article |
| 2023 | North-America | Identification of Novel Genetic Risk Factors for Focal Segmental Glomerulosclerosis in Children: Results From the Chronic Kidney Disease in Children (CKiD) Cohort | Durand A, Winkler CA, Vince N, Douillard V, Geffard E, Binns-Roemer E, Ng DK, Gourraud PA, Reidy K, Warady B, Furth S, Kopp JB, Kaskel FJ, Limou S. | Am J Kidney Dis | 36623684 | Research Article |
| 2023 | North-America | Clinical and Genetic Characteristics of CKD Patients with High-Risk APOL1 Genotypes | Elliott MD, Marasa M, Cocchi E, Vena N, Zhang JY, Khan A, Krishna Murthy S, Bheda S, Milo Rasouly H, Povysil G, Kiryluk K, Gharavi AG. | J Am Soc Nephrol | 36758113 | Research Article |
| 2023 | North-America | Kidney Disease Progression in Membranous Nephropathy among Black Participants with High-Risk APOL1 Genotype | Chen DP, Henderson CD, Anguiano J, Aiello CP, Collie MM, Moreno V, Hu Y, Hogan SL, Falk RJ; CureGN. | Clin J Am Soc Nephrol | 36763808 | Research Article |
| 2023 | North-America | Rapid Progression of Focal Segmental Glomerulosclerosis in Patients with High-Risk APOL1 Genotypes | Kallash M, Wang Y, Smith A, Trachtman H, Gbadegesin R, Nester C, Canetta P, Wang C, et al. | Clin J Am Soc Nephrol | 36763813 | Research Article |
| 2023 | North-America | The role of HLA antigens in recurrent primary focal segmental glomerulosclerosis | Batal I, Khairallah P, Weins A, Andeen NK, Stokes MB. | Front Immunol | 36911713 | Research Article |
| 2023 | North-America | Inaxaplin for Proteinuric Kidney Disease in Persons with Two APOL1 Variants | Egbuna O, Zimmerman B, Manos G, Fortier A, Chirieac MC, Dakin LA, Friedman DJ, Bramham K, et al. | N Engl J Med | 36920755 | Research Article |
| 2023 | North-America | APOL1 and the risk of adverse renal outcomes in patients of African ancestry with systemic lupus erythematosus | Chung CP, Karakoc G, Dickson A, Liu G, Gamboa JL, Mosley JD, Cox NJ, Kawai VK. | Lupus | 37105192 | Research Article |
| 2023 | North-America | CMV-associated collapsing focal segmental glomerulosclerosis after kidney transplant in a pediatric patient | Parr MFE, Hidalgo G, Goldstein MJ, Batal I, Lieberman KV, Amoruso MR, Baer AZ, Jain NG. | Pediatr Transplant | 37128132 | Research Article |
| 2023 | North-America | Implementation of a culturally competent APOL1 genetic testing programme into living donor evaluation: A two-site, non-randomised, pre-post trial design | Smith JD, Agrawal A, Wicklund C, Duquette D, Friedewald J, Rasmussen LV, Gacki-Smith J, Tandon SD, Muhammad LN, Yancy CW, Dong S, Cooper M, Gilbert A, Shetty A, Gordon EJ. | BMJ Open | 37188469 | Research Article |
| 2023 | North-America | Polygenic risk affects the penetrance of monogenic kidney disease | Khan A, Shang N, Nestor JG, Weng C, Hripcsak G, Harris PC, Gharavi AG, Kiryluk K. | medRxiv | 37214819 | Research Article |
| 2023 | North-America | Dietary Patterns, Apolipoprotein L1 Risk Genotypes, and CKD Outcomes Among Black Adults in the Reasons for Geographic and Racial Differences in Stroke (REGARDS) Cohort Study | Ilori TO, Brooks MS, Desai PN, Cheung KL, Judd SE, Crews DC, Cushman M, Winkler CA, Shlipak MG, Kopp JB, Naik RP, Estrella MM, Gutiérrez OM, Kramer H. | Kidney Med | 37229446 | Research Article |
| 2023 | North-America | APOL1 promotes endothelial cell activation beyond the glomerulus | Carracedo M, Ericson E, Ågren R, Forslöw A, Madeyski-Bengtson K, Svensson A, Riddle R, Christoffersson J, et al. | iScience | 37250770 | Research Article |
| 2023 | North-America | Early recurrence of focal segmental glomerulosclerosis in a kidney transplant recipient with APOL1 one risk variant | Acharya R, Upadhyay K. | BMJ Case Rep | 37258049 | Research Article |
| 2023 | North-America | Genome-Wide Association Study of CKD Progression | Robinson-Cohen C, Triozzi JL, Rowan B, He J, Chen HC, Zheng NS, Wei WQ, Wilson OD, Hellwege JN, Tsao PS, Gaziano JM, Bick A, Matheny ME, Chung CP, Lipworth L, Siew ED, Ikizler TA, Tao R, Hung AM. | J Am Soc Nephrol | 37261792 | Research Article |
| 2023 | North-America | Association of Recipient APOL1 Kidney Risk Alleles With Kidney Transplant Outcomes | Roy N, Morales-Alvarez MC, Anis KH, Goral S, Doria C, Kopp JB, Winkler CA, Feng R, Rosas SE. | Transplantation | 37527489 | Research Article |
| 2023 | North-America | African American/Black race, apolipoprotein L1 , and serum creatinine among persons with HIV | Shelton BA, Sawinski D, Peter I, Maclennan PA, Pelletier NF, Nadkarni G, Julian B, Saag M, et al. | AIDS | 37650767 | Research Article |
| 2023 | North-America | Genetic Inhibition of APOL1 Pore-Forming Function Prevents APOL1-Mediated Kidney Disease | Hung AM, Assimon VA, Chen HC, Yu Z, Vlasschaert C, Triozzi JL, Chan H, Wheless L, et al. | J Am Soc Nephrol | 37798822 | Research Article |
| 2023 | North-America | Association between APOL1 risk variants and the occurrence of sepsis in Black patients hospitalized with infections: a retrospective cohort study | Jiang L, Liu G, Oeser A, Ihegword A, Dickson AL, Daniel LL, Hung AM, Cox NJ, Chung CP, Wei WQ, Stein CM, Feng Q. | Elife | 37882666 | Research Article |
| 2023 | North-America | Unveiling APOL1 Haplotypes: A Novel Classification Through Probe-Independent Quantitative Real-Time PCR | Dogan M, Watkins C, Ingram H, Moore N, Rucker GM, Gower EG, Eason JD, Bhalla A, Talwar M, Nezakatgoo N, Eymard C, Helmick R, Vanatta J, Bajwa A, Kuscu C, Kuscu C. | bioRxiv | 37905084 | Research Article |
| 2023 | North-America | Local Inflammation But Not Kidney Cell Infection Associated with High APOL1 Expression in COVID-Associated Nephropathy | Nguyen JK, Wu Z, Agudelo J, Herlitz LC, Miller AW, Bruggeman LA. | Kidney360 | 37927001 | Research Article |
| 2023 | North-America | Strong protective effect of the APOL1 p.N264K variant against G2-associated focal segmental glomerulosclerosis and kidney disease | Gupta Y, Friedman DJ, McNulty MT, Khan A, Lane B, Wang C, Ke J, Jin G, Wooden B, Knob AL, Lim TY, et al. | Nat Commun | 38036523 | Research Article |
| 2023 | North-America | CR1 variants contribute to FSGS susceptibility across multiple populations | Skitchenko R, Modrusan Z, Loboda A, Kopp JB, Winkler CA, Sergushichev A, Gupta N, Stevens C, Daly MJ, Shaw A, Artomov M. | medRxiv | 38076851 | Research Article |
| 2023 | South-America | APOL1 Gene Variants and Risk for Cardiovascular Disease | Duran CE, Estacio M, Espinosa D, Manzi E, Posada JG, Mesa L, Schweineberg J. | Kidney Blood Press Res | 37778341 | Research Article |
| 2024 | Africa | Association of Normal and Mutated APOL1 G2 Rs60910145 alleles with SCD, Body Mass Index, and Renal Function Biomarkers and Indices | Ismail AM, Nour BM, Abakar AD, Almugadam BS, Altayb HN, Ahmed RT, Elkarsany MEM. | Curr Res Transl Med | 38246019 | Research Article |
| 2024 | Africa | Proteomic insights into the pathophysiology of hypertension-associated albuminuria: Pilot study in a South African cohort | Govender MA, Stoychev SH, Brandenburg JT, Ramsay M, Fabian J, Govender IS. | Clin Proteomics | 38402394 | Research Article |
| 2024 | Africa | APOL1 Bi- and Monoallelic Variants and Chronic Kidney Disease in West Africans | Gbadegesin RA, Ulasi I, Ajayi S, Raji Y, Olanrewaju T, Osafo C, Ademola AD, Asinobi A,et al. | N Engl J Med | 39465900 | Research Article |
| 2024 | Africa | G1 and G2 variants of apolipoprotein L1 among Central African population in Trypanosoma brucei gambiense endemic rural area | Mupepe DM, Wameso MN, Situakibanza HN, Ekulu PM, Makulo JRR, Kayembe JMN, Nkoy AB, et al. | J Infect Dev Ctries | 39616492 | Research Article |
| 2024 | Europe | Phenome-wide analysis reveals epistatic associations between APOL1 variants and chronic kidney disease and multiple other disorders | Adamson WE, Noyes H, Johnson P, Cooper A, Monckton DG, Ogunsola J, Beckett-Hill G, Sullivan M, Mark P, Parekh RS, MacLeod A. | EBioMedicine | 38360481 | Research Article |
| 2024 | Nigeria | Sickle cell trait, APOL1 risk allele status and chronic kidney disease among ART-experienced adults living with HIV in northern Nigeria | Abdulhamid A, Shepherd BE, Wudil UJ, Van Wyk C, Dankishiya FS, Hussaini N, Wester CW, Aliyu MH. | Int J STD AIDS | 38915133 | Research Article |
| 2024 | North-America | Genome-Wide Polygenic Risk Score for CKD in Individuals with APOL1 High-Risk Genotypes | Vy HMT, Coca SG, Sawant A, Sakhuja A, Gutierrez OM, Cooper R, Loos RJF, Horowitz CR, Do R, Nadkarni GN. | Clin J Am Soc Nephrol | 37962879 | Research Article |
| 2024 | North-America | Safety and Tolerability of the APOL1 Inhibitor, Inaxaplin, following Single- and Multiple-Ascending Doses in Healthy Adults | Egbuna O, Audard V, Manos G, Tian S, Hagos F, Chertow GM. | Glomerular Dis | 38600955 | Research Article |
| 2024 | North-America | Unveiling APOL1 haplotypes in a predominantly African-American cohort of kidney transplant patients: a novel classification using probe-independent quantitative real-time PCR | Dogan M, Watkins C, Ingram H, Moore N, Rucker GM, Gower EG, Eason JD, Bhalla A, Talwar M, Nezakatgoo N, Eymard C, Helmick R, Vanatta J, Bajwa A, Kuscu C, Kuscu C. | Front Med (Lausanne) | 38660426 | Research Article |
| 2024 | North-America | Incorporating genetic testing into a routine kidney clinic | Zhang J, Zeng W, Hamrahian S, Maarouf OH. | Clin Nephrol | 38660889 | Research Article |
| 2024 | North-America | African ancestry-derived APOL1 risk genotypes show proximal epigenetic associations | Breeze CE, Lin BM, Winkler CA, Franceschini N. | BMC Genomics | 38714935 | Research Article |
| 2024 | North-America | Association of Preterm Birth with Adverse Glomerular Disease Outcomes in Children and Adults | Isaac J, Troost J, Wang Y, Garrity K, Kaskel F, Gbadegesin R, Reidy K. | Clin J Am Soc Nephrol | 38728081 | Research Article |
| 2024 | North-America | Association of Preterm Birth with Adverse Glomerular Disease Outcomes in Children and Adults | Isaac JS, Troost JP, Wang Y, Garrity K, Kaskel F, Gbadegesin R, Reidy KJ. | Clin J Am Soc Nephrol | 38728081 | Research Article |
| 2024 | North-America | Focal Segmental Glomerulosclerosis Patient Baseline Characteristics in the Sparsentan Phase 3 DUPLEX Study | Trachtman H, Radhakrishnan J, Rheault MN, Alpers CE, Barratt J, Heerspink HJL, Noronha IL, Perkovic V, et al. | Kidney Int Rep | 38765567 | Research Article |
| 2024 | North-America | APOL1 Kidney Risk Variants and Long-Term Kidney Function in Healthy Middle-Aged Black Individuals: The Atherosclerosis Risk in Communities (ARIC) Study | Doshi MD, Li L, Naik AS, Thomas CP. | Kidney Med | 38799783 | Research Article |
| 2024 | North-America | Diagnostic Yield of APOL1 p.N264K Variant Screening in Daily Practice | Narjoz C, Vinh-Hoang-Lan Julie Tran, Rabant M, Karras A, Pallet N. | Kidney Int Rep | 38899219 | Research Article |
| 2024 | North-America | Protein-truncating variant in APOL3 increases chronic kidney disease risk in epistasis with APOL1 risk alleles | Zhang DY, Levin MG, Duda JT, Landry LG, Witschey WR, Damrauer SM, Ritchie MD, Rader DJ. | JCI Insight | 39163132 | Research Article |
| 2024 | North-America | Increased risk of kidney failure in patients with genetic kidney disorders | Elliott MD, Vena N, Marasa M, Cocchi E, Bheda S, Bogyo K, Shang N, Zanoni F, et al. | J Clin Invest | 39225089 | Research Article |
| 2024 | North-America | CRISPR-enabled point-of-care genotyping for APOL1 genetic risk assessment | Greensmith R, Lape IT, Riella CV, Schubert AJ, Metzger JJ, Dighe AS, Tan X, Hemmer B, et al. | EMBO Mol Med | 39271961 | Research Article |
| 2024 | North-America | Prevalence of Mendelian Kidney Disease Among Patients With High-Risk APOL1 Genotypes Undergoing Commercial Genetic Testing in the United States | da Silva Francisco R Jr, Punj S, Vincent L, Sanapareddy N, Bhalla V, Chertow GM, Keen-Kim D, Charu V. | Kidney Int Rep | 39291188 | Research Article |
| 2024 | South-America | Idiopathic collapsing glomerulopathy is associated with APOL1 high-risk genotypes or Mendelian variants in most affected individuals in a highly admixed population | Neves PD, Watanabe A, Watanabe EH, Narcizo AM, Nunes K, Lerario AM, Ferreira FM, Cavalcante LB, et al. | Kidney Int | 38143038 | Research Article |
| 2024 | South-America | Steroid-Resistant Nephrotic Syndrome Is Associated With a Unique Genetic Profile in a Highly Admixed Pediatric Population | Watanabe A, Miranda de Menezes Neves PD, Nunes K, Lerario AM, Watanabe EH, Ferreira FM, Avancini Costa Malheiros DM, et al. | Kidney Int Rep | 39698360 | Research Article |
| 2024 | Africa | Association of Normal and Mutated APOL1 G2 Rs60910145 alleles with SCD, Body Mass Index, and Renal Function Biomarkers and Indices | Ismail AM, Nour BM, Abakar AD, Almugadam BS, Altayb HN, Ahmed RT, Elkarsany MEM. | Curr Res Transl Med | 38246019 | Research Article |
| 2024 | Europe | Phenome-wide analysis reveals epistatic associations between APOL1 variants and chronic kidney disease and multiple other disorders | Adamson WE, Noyes H, Johnson P, Cooper A, Monckton DG, Ogunsola J, Beckett-Hill G, Sullivan M, Mark P, Parekh RS, MacLeod A. | EBioMedicine | 38360481 | Research Article |
| 2024 | North-America | Genome-Wide Polygenic Risk Score for CKD in Individuals with APOL1 High-Risk Genotypes | Vy HMT, Coca SG, Sawant A, Sakhuja A, Gutierrez OM, Cooper R, Loos RJF, Horowitz CR, Do R, Nadkarni GN. | Clin J Am Soc Nephrol | 37962879 | Research Article |
| 2024 | North-America | Safety and Tolerability of the APOL1 Inhibitor, Inaxaplin, following Single- and Multiple-Ascending Doses in Healthy Adults | Egbuna O, Audard V, Manos G, Tian S, Hagos F, Chertow GM. | Glomerular Dis | 38600955 | Research Article |
| 2024 | North-America | Unveiling APOL1 haplotypes in a predominantly African-American cohort of kidney transplant patients: a novel classification using probe-independent quantitative real-time PCR | Dogan M, Watkins C, Ingram H, Moore N, Rucker GM, Gower EG, Eason JD, Bhalla A, Talwar M, Nezakatgoo N, Eymard C, Helmick R, Vanatta J, Bajwa A, Kuscu C, Kuscu C. | Front Med (Lausanne) | 38660426 | Research Article |
| 2024 | North-America | Incorporating genetic testing into a routine kidney clinic | Zhang J, Zeng W, Hamrahian S, Maarouf OH. | Clin Nephrol | 38660889 | Research Article |
| 2024 | North-America | African ancestry-derived APOL1 risk genotypes show proximal epigenetic associations | Breeze CE, Lin BM, Winkler CA, Franceschini N. | BMC Genomics | 38714935 | Research Article |
| 2024 | North-America | Association of Preterm Birth with Adverse Glomerular Disease Outcomes in Children and Adults | Isaac J, Troost J, Wang Y, Garrity K, Kaskel F, Gbadegesin R, Reidy K. | Clin J Am Soc Nephrol | 38728081 | Research Article |
| 2024 | North-America | APOL1 Kidney Risk Variants and Long-Term Kidney Function in Healthy Middle-Aged Black Individuals: The Atherosclerosis Risk in Communities (ARIC) Study | Doshi MD, Li L, Naik AS, Thomas CP. | Kidney Med | 38799783 | Research Article |
| 2024 | North-America | Diagnostic Yield of APOL1 p.N264K Variant Screening in Daily Practice | Narjoz C, Vinh-Hoang-Lan Julie Tran, Rabant M, Karras A, Pallet N. | Kidney Int Rep | 38899219 | Research Article |
| 2024 | North-America | Protein-truncating variant in APOL3 increases chronic kidney disease risk in epistasis with APOL1 risk alleles | Zhang DY, Levin MG, Duda JT, Landry LG, Witschey WR, Damrauer SM, Ritchie MD, Rader DJ. | JCI Insight | 39163132 | Research Article |
| 2024 | North-America | Increased risk of kidney failure in patients with genetic kidney disorders | Elliott MD, Vena N, Marasa M, Cocchi E, Bheda S, Bogyo K, Shang N, Zanoni F, Verbitsky M, Wang C, Kolupaeva V, Jin G, Sofer M, Gras Pena R, Canetta PA, Bomback AS, Guay-Woodford LM, Hou J, Gillespie BW, Robinson BM, Klein JB, Rheault MN, Smoyer WE, Greenbaum LA, Holzman LB, Falk RJ, Parsa A, Sanna-Cherchi S, Mariani LH, Kretzler M, Kiryluk K, Gharavi AG; CureGN Consortium. | J Clin Invest | 39225089 | Research Article |
| 2024 | North-America | Prevalence of Mendelian Kidney Disease Among Patients With High-Risk APOL1 Genotypes Undergoing Commercial Genetic Testing in the United States | da Silva Francisco R Jr, Punj S, Vincent L, Sanapareddy N, Bhalla V, Chertow GM, Keen-Kim D, Charu V. | Kidney Int Rep | 39291188 | Research Article |
| 2025 | North-America | Diabetes mellitus and APOL1 genotype increase the risk for chronic kidney disease progression in sickle cell disease | Sun R, Srivastava A, Zapater JL, Njoku F, Han J, Ivy Z, Farooqui M, Rizvi I, Layden B, Molokie RE, Gordeuk VR, Saraf SL. | Blood | 40112283 | Research Article |
| 2025 | Africa | APOL1 genotype and patient outcomes in US and South African transplant recipients with HIV who received kidneys from donors with HIV | Freercks R, Rodrigues M, Manning K, Heymann J, Kopp JB, Nagiah S, Rana M, Florman S, Friedman-Moraco R, Stock P, Gilbert A, Mehta S, Stosor V, Pereira MR, Morris MI, Hand J, Haidar G, Malinis M, Santos CAQ, Schaenman J, Blumberg EA, Wojciechowski D, Odim J, Massie A, Selvan MT, Bagnasco S, Segev D, Tobian AA, Muller E, Durand CM, Redd AD. | medRxiv | 40321250 | Research Article |
| 2025 | South-America | Minimal Change Disease and Focal Segmental Glomerulosclerosis Are Associated with Good Kidney Prognosis and Thrombotic Microangiopathy with Poor Kidney Survival in Patients with COVID-19-Associated Nephropathies | Neves PDMM, Araújo SA, Wanderley DC, Sodré AM, Campos MAG, Watanabe EH, Malheiros DMAC, Cavalcante LB, Ledesma FL, Israel KCP, Barcelos FL, Pinho LLF, Barbosa FAM, Miranda SMC, Dantas M, Britto WLM, Paixão JO, Madeira RL, Guedes FL, Luchi WM, Cuvello-Neto AL, Marques IDB, Almeida JB, Vasco RFV, Monteiro A, Ladchumananandasivam FR, Júnior AALT, Gomes OV, Hagemann R, Ferra-Neto OA, Santos RF, Costa DMDN, Luz-Neto ER, Soares LCBA, Mubarac RS, Costa KMAH, Weissheimer R, Almeida CM, da Silva BAB, Miranda LS, Iannuzzi LR, Murata GM, Noronha IL, Salgado-Filho N, Onuchic LF, Silva GEB. | Am J Nephrol | 39662073 | Research Article |
| 2025 | South-America | Carrying APOL1 G1 allele is associated with cardiovascular complications during COVID−19 in an admixed population | Nathan A. Cadore, Bibiana S. de O. Fam, Giovanna C. Giudicelli, Thayne W. Kowalski, Renan C. Sbruzzi, Marcos A. Castro e Silva, Marilea F. Feira, Célia M. B. Souza, Dirceu R. da Silva, Osvaldo Artigalás, Renan B. Lemes, Maíra R. Rodrigues, Kelly Nunes, Alexandre C. Pereira, Lygia V. Pereira, Tábita Hünemeier, Fernanda S. L. Vianna | Human Genomics | 40887632 | Research Article |
| 2025 | South-America | APOL1 G1/G2 variants associated with CKD in Afro-Brazilians | Célia M. B. Souza, Bibiana S. O. Fam, Giovanna C. Giudicelli, Nathan A. Cadore, Mariléa F. Feira, Mayara J. Prado, Thayne W. Kowalski, Osvaldo A. P. Artigalás, Renan B. Lemes, Maíra R. Rodrigues, Mauro R. S. Junior, William C. Silva, Maicon D. Torely, Franciele M. Barbosa, Alexandre C. Pereira, Lygia V. Pereira, Tábita Hünemeier, Francisco V. Veronese, Fernanda S. L. Vianna | Kidney International Reports | PII: S246802492500600X | Research Article |
| 2025 | Africa | De novo rates of a Trypanosoma-resistant mutation in two human populations | Melamed D, Shemer R, Bolotin E, Yakass MB, Fink-Barkai D, Hiadzi EK, Skorecki KL, Livnat A. | Proc Natl Acad Sci U S A | 40854136 | Research Article |
| 2025 | Europe | Quantifying APOL1, Human Leukocyte Antigen, and Other Genetic Contributions to Unexplained Kidney Failure | Sadeghi-Alavijeh O, Chan MMY, Tzoumkas K, Doctor GT, Gale DP. | Am J Kidney Dis | 40967557 | Research Article |
| 2025 | Europe | APOL1 risk variants induce metabolic reprogramming of podocytes in patient-derived kidney organoids | Song H, Dumas SJ, Wang G, Ma L, Witjas F, Avramut MC, van den Berg CW, Rocco MV, Freedman BI, Rabelink TJ, Spijker HS. | Stem Cell Reports | 41043427 | Research Article |
| 2025 | North-America | Comparing adolescent glomerular disease clinical outcomes to the clinical outcomes in childhood, young adult, and adult-onset glomerular disease in the CureGN database | Garrity K, Putnam N, Kamil ES, Massengill S, Khalid M, Srivastava R, Isaacs J, Salmon E. | Pediatr Nephrol | 39729127 | Research Article |
| 2025 | North-America | APOL1-Risk Genotype Induces Inflammatory and Hypoxic Gene Expression in Donor Kidneys | Unes M, Kolli S, Mehta S, Manithody C, Bruno J, Lentine KL, Jain A, Nazzal M, Caliskan Y. | Genes (Basel) | 41010023 | Research Article |
| 2025 | North-America | Development of Polygenic Risk Score for Persistent Albuminuria in Children and Adults With Sickle Cell Anemia | Zahr RS, Kang G, Zhang X, Rashkin SR, Kovesdy CP, Takemoto C, Weiss M, Lebensburger J, Ataga KI, Saraf SL. | Am J Hematol | 40186439 | Research Article |
| 2025 | North-America | Small molecule APOL1 inhibitors as a precision medicine approach for APOL1-mediated kidney disease | Zimmerman B, Dakin LA, Fortier A, Nanou E, Blasio A, Mann J, Miller H, Fletcher M, Wang T, Nanthakumar S, McCarthy G, Matar C, Matsye P, Wang G, Snyder P, Daniel K, Swamy H, Sullivan K, Bright F, Powers A, Gagnon KJ, Lu F, Paula S, Khare-Pandit S, Henry L, Hamel M, Denis F, Nicolas O, Hariparsad N, Kumar S, Proctor J, Senter T, Furey B, Bunnage ME. | Nat Commun | 39747090 | Research Article |
| 2025 | North-America | The Diagnostic Yield of Genomic Sequencing-based Genetic Kidney Disease Testing in Kidney Transplant Candidates: Experience at an Urban US Transplant Center | Abu Al Rub F, Elsurer Afsar R, Fleetwood VA, Bastani B, Randall H, Nazzal M, Varma C, Afsar B, Jackson H, Yount S, Wooley C, Light J, Davis V, Caliskan Y, Lentine KL. | Transplantation | 39819994 | Research Article |
| 2025 | North-America | The APOL1 p.N264K variant is co-inherited with the G2 kidney disease risk variant through a proximity recombination event | Simeone CA, McNulty MT, Gupta Y, Genovese G, Sampson MG, Sanna-Cherchi S, Friedman DJ, Pollak MR. | G3 (Bethesda) | 39658338 | Research Article |
| 2025 | North-America | Genetic Assessment of Living Kidney Transplant Donors: A Survey of Canadian Practices | Zahran S, Bei KF, Adil A, Okoh P, Kitzler T, Alam A. | Can J Kidney Health Dis | 39802879 | Research Article |
| 2025 | North-America | A Human Glomerular Disease Atlas defines the APOL1-JAK-STAT feed forward loop in focal segmental glomerulosclerosis | McCown PJ, Boys C, Datta S, Nair V, Otto E, Fermin D, Hartman J, Annese F, Alakwaa F, Latt KZ, Smith C, Godfrey B, Larkina M, Ashley-Koch AE, Garrett ME, Moore S, Soldano K, Alaba M, Mariani LH, Naik AS, Fallegger R, Eddy S, Saez-Rodriguez J, Olabisi OA, Kretzler M; Nephrotic Syndrome Study Network (NEPTUNE). | medRxiv | 41001468 | Research Article |
| 2025 | North-America | Genomewide Screen Identifies Peroxisomal Role in APOL1 Podocytopathy | Kim J, Karel IZ, Song H, Dewalt M, Orwick S, Buelow DR, Lee K, Brodsky SV, Blissett A, Cocucci E, Baker SD, Lin PH, Pabla NS, Madhavan SM. | medRxiv | 40034797 | Research Article |
